# A Cost‐Effective Computational Strategy for the Electronic Layout Characterization of a Second Generation Light‐Driven Molecular Rotary Motor in Solution

**DOI:** 10.1002/jcc.70023

**Published:** 2025-01-11

**Authors:** Raoul Carfora, Federico Coppola, Paola Cimino, Alessio Petrone, Nadia Rega

**Affiliations:** ^1^ Scuola Superiore Meridionale Napoli Italy; ^2^ Department of Chemical Sciences University of Napoli Federico II, Complesso Universitario di M.S. Angelo Napoli Italy; ^3^ Istituto Nazionale Di Fisica Nucleare sezione di Napoli, Complesso Universitario di M.S. Angelo Napoli Italy

**Keywords:** cost‐effective computations, excited states, light‐driven molecular rotors, solvation, TDDFT

## Abstract

Light‐driven molecular rotary motors are nanometric machines able to convert light into unidirectional motions. Several types of molecular motors have been developed to better respond to light stimuli, opening new avenues for developing smart materials ranging from nanomedicine to robotics. They have great importance in the scientific research across various disciplines, but a detailed comprehension of the underlying ultrafast photophysics immediately after photo‐excitation, that is, Franck–Condon region characterization, is not fully achieved yet. For this aim, it is first required to rely on an accurate description at *ab initio* level of the system in this potential energy region before performing any further step, that is, dynamics. Thus, we present an extensive investigation aimed at accurately describing the electronic structure of low‐lying electronic states (electronic layout) of a molecular rotor in the Franck–Condon region, belonging to the class of overcrowded alkenes: 9‐(2‐methyl‐2,3‐dihydro‐1H‐cyclopenta[a]naphthalen‐1‐ylidene)‐9H‐fluorene. This system was chosen since its photophysics is very interesting for a more general understanding of similar compounds used as molecular rotors, where low‐lying electronic states can be found (whose energetic interplay is crucial in the dynamics) and where the presence of different substituents can tune the HOMO‐LUMO gap. For this scope, we employed different theory levels within the time‐dependent density functional theory framework, presenting also a careful comparison adopting very accurate post Hartree–Fock methods and characterizing also the different conformations involved in the photocycle. Effects on the electronic layout of different functionals, basis sets, environment descriptions, and the role of the dispersion correction were all analyzed in detail. In particular, a careful treatment of the solvent effects was here considered in depth, showing how the implicit solvent description can be accurate for excited states in the Franck–Condon region by testing both linear‐response and state‐specific formalisms. As main results, we chose two cost‐effective (accurate but relatively cheap) theory levels for the ground and excited state descriptions, and we also verified how choosing these different levels of theory can influence the curvature of the potential via a frequency analysis of the normal modes of vibrations active in the Raman spectrum. This theoretical survey is a crucial step towards a feasible characterization of the early stage of excited states in solution during photoisomerization processes wherein multiple electronic states might be populated upon the light radiation, leading to a future molecular‐level interpretation of time‐resolved spectroscopies.

## Graphical abstract

Cutting‐edge computational techniques, such as TD‐DFT and multireference methods, are here gauged for the accurate description of the electronic layout of light‐driven molecular rotary motors.

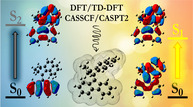

## 1 Introduction

**FIGURE 1:**
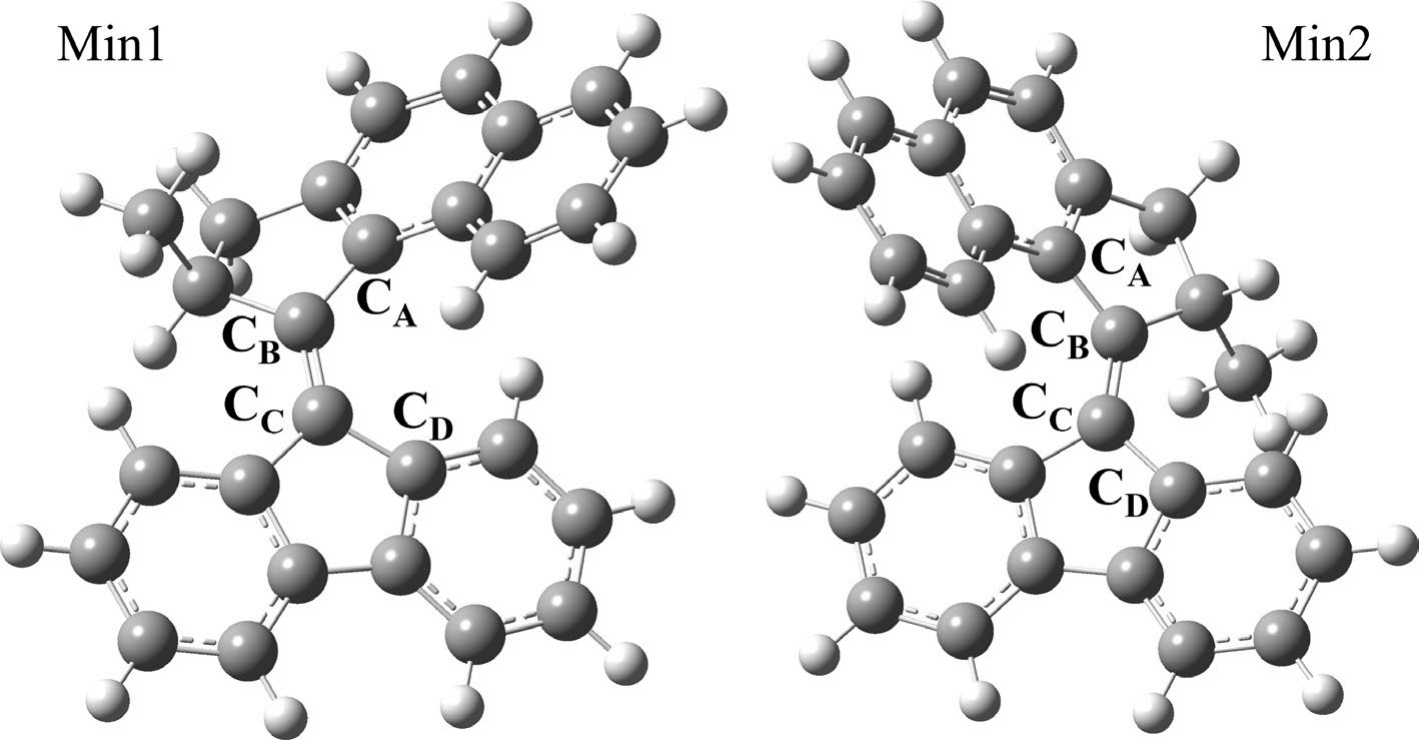
B3LYP/6‐31G(d,p)/C‐PCM 9‐(2‐methyl‐2,3‐dihydro‐1H‐cyclopenta[a]naphthalen‐1‐ylidene)‐9H‐fluorene minimum energy structures in cyclohexane solution. The stable (Min1, left panel) and unstable (Min2, right panel) isomers are both reported. The fluorene moiety corresponds to the stator while the naphthalene moiety to the rotor. Hydrogen and carbon atoms are represented in white and light grey, respectively.

Artificial molecular machines represent an active and promising research field closely linked to the development of new materials, in which the translational or rotational movement of a molecular engine, controlled using external stimuli, can be used for several technological applications [1, 2, 3, 4, 5, 6, 7, 8]. Different classes of molecular motors, operated with different external energy sources, were developed in the last three decades [4, 5, 9, 10]. An elegant way to control these systems is using the electromagnetic radiation; an example of molecules of this type are the so‐called light‐driven molecular rotary motors (LDMRMs) developed for the first time by Feringa and co‐workers [11]. These molecules are composed of two parts, one called a rotor (free to move) and another one called stator (in our case, the fluorene moiety, which in applications can be linked to a surface or to a rigid structure), that are connected together by a double bond (see Figure 1). The LDMRMs can perform an unidirectional rotation around a double bond in a four step cycle. In the first step, the photoisomerization, the molecule starting from its most stable conformation (see Min1, Figure 1), after absorbing the light, evolves in an excited electronic state and returns, after crossing one or more conical intersection(s), to the ground state as an unstable isomer (see Min2, Figure 1). In the second step, called thermal helix inversion, this unstable isomer is converted into a more stable conformation using the thermal energy. Repeating these two steps, the molecule completes the entire cycle of rotation. We point out that the two thermal steps represent the bottleneck of the cycle, and therefore they limit the rotation frequency of these systems. Over the years, different approaches were developed to overcome this limitation. For example, Frutos et al. [12] starting from a photoactive molecular switch and introducing the chiral environment with appropriate hydrogen bonds, designed a retinal‐based motor in which the cycle of rotation is characterized only by two photochemical steps, without the need for thermal steps. Moreover, more recently, Durbeej et al. [13] explored the possibility of using isotopic substitution of hydrogen atoms to introduce the chirality needed to have a unidirectional rotary motion in a light‐driven molecular motor.

We propose here a theoretical‐computational study towards an accurate and cost‐effective *ab initio*characterization of the potential energy region involved immediately after the photo‐excitation, called the Franck–Condon (FC) region, towards the study of the early stage of excited states in solution taking part in the photo‐cycle. As a matter of fact, during the photoisomerization process, multiple electronic states might be populated upon the light irradiation, and it is crucial to simultaneously describe them with high accuracy. Such a preliminary step is mandatory for providing a molecular‐level interpretation of related time‐resolved experiments. For this aim, it is first required to rely on an accurate and cost‐effective description at the *ab initio* level of the system in the FC region before performing any further step, that is, dynamics. Thus, in this work we present an extensive investigation aimed at accurately describing the electronic structure of low‐lying singlet excited states (electronic layout) of an LDMRM belonging to the second generation of molecular rotors (see References [5] and [14] for a detailed classification), an overcrowded alkene: 9‐(2‐methyl‐2,3‐dihydro‐1H‐cyclopenta[a]naphthalen‐1‐ylidene)‐9H‐fluorene, namely Cyclo‐NYF and reported in Figure 1. In particular, second‐generation rotors present a similar photodynamics, where the central bond elongates and decreases in bond order, allowing free rotation about the axle as the molecule traverses the first singlet (S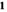) potential energy surface. From the optically bright Franck–Condon state, the motor undergoes an ultrafast barrierless (or almost barrierless) relaxation on the order of a few hundred fs towards the region of the global minimum of the excited state, usually “dark” and with a relatively longer excited state lifetime [15, 16]. However, the exact origin and structure of this dark state remain unclear, especially in the presence of substituents with different electronic features installed on the motor. Studying the presented system can have a broader impact on the understanding of this process, and Cyclo‐NYF is here chosen since there are several experimental (and few computational) works where it is well understood that the system evolves in the FC on a single potential surface, with no double excitations involved [16, 17, 18, 19, 20, 21, 22, 23, 24, 25, 26]. Such 2nd generation molecular rotors can have different excited states involved in the photophysics, although the chosen system evolves adiabatically on the first excited state upon excitation during the first hundreds of fs and then fully isomerizes crossing a CI [14, 20]. Moreover, its photophysics is very interesting for a more general understanding of similar compounds used as molecular rotors, where low‐lying electronic states can be found (localized on both or one half of the system versus charge transfer states), and since their HOMO‐LUMO gap can be tuned by extending the π‐system to increase conjugation of the chromophore [14].

From a theoretical point of view, the complete description of the photophysical pathway of such phenomena might require multireference methods [27] or density functional theory (DFT) and its time‐dependent version [28, 29, 30, 31] in the mixed‐reference spin‐flip (MRSF‐TDDFT) variant [16, 32]. The latter method, or even more computationally demanding ones, that is, the extended multi‐state multi‐reference perturbation theories [33], would be useful in the proximity of the conical intersection among the electronic states to give its correct topology [34]. On another hand, since we are interested in performing a preliminary step for describing the early stage of this photoinduced process in the FC region, we used canonical TD‐DFT for the *ab initio* treatment of the system, since it has an optimal balance between accuracy and computational cost, and, in its hybrid version, it has been vastly used for the theoretical characterization of both vibrational and dynamical properties of molecules [35, 36, 37, 38, 39, 40, 41, 42, 43, 44] and the description of the electronic structure of both the ground and excited electronic states in macro‐molecular systems of material [45, 46, 47, 48, 49, 50, 51, 52, 53, 54, 55, 56, 57, 58, 59] or biological [60, 61, 62, 63, 64, 65, 66, 67, 68, 69] interest. Since canonical TDDFT might face issues in the proximity of the FC region, when excited states exhibit a significant double excitation character [70, 71, 72], we also tested multi‐configurational second‐order perturbation theory to be sure that single excitations are the most relevant for the system and canonical TD‐DFT could still be used. As for a more general discussion, the choice of the computational approach to be used, can be made by performing a preliminary step by testing different levels of theory, monitoring, if possible, more than one parameter and/or property of interest, to find the right compromise between accuracy and computational cost. Such a strategy can sometimes lead to a fortuitous agreement with experiments, but for that is, an error cancellation. In this work, our main goal is to stress that it is even more important to have a robust internal standard for the accuracy of the level of theory (i.e., post‐HF methods) to be used as a comparison. As an additional note, not only in the minimum energy geometry but also in several regions of the PES (i.e., around the FC region, at least for ultrafast dynamics), it is also crucial to gauge the theory level. Time‐resolved experiments, such as transient absorption and femtosecond stimulated Raman spectroscopy, can offer valuable insights to guide and gauge computations, promoting a synergistic strategy to enhance both the accuracy and the molecular understanding of the modeling strategy, particularly the ultrafast evolution of the system following photoexcitation in the FC region. Moreover, it is not a trivial task to accurately describe the multiple low‐lying excited states that can be potentially involved in the relaxation pathway, where different excitation characters (i.e., localized and/or charge transfer, CT) need to be simultaneously described in a cheap but still accurate way. Thus, we also focused on the effect of the surrounding environment on the description of different electronic states relevant in the FC region. We first ensured that the solvent effects could be included by employing a cost‐effective implicit solvent description, here shown to be accurate for excited states in the FC region, by testing also different formalisms for the implicit solvent response in the excited states. Indeed, the implicit solvent model was exploited for predicting the vertical transition energies, comparing the results of two different formalisms, that are either the Linear Response (LR) formulation [30, 73, 74], or the so‐called State‐Specific (SS) [75, 76, 77, 78] approach. Thus, we present an extensive study towards the correct description of the system electronic layout of the low‐lying electronic states, gauging different levels of theory rooted in the DFT framework, and presenting also a careful comparison employing very accurate post Hartree–Fock methods.

We believe that this study is a fundamental preliminary step for the accurate molecular characterization of the photoisomerization step, in which the molecule, after interacting with the electromagnetic radiation, evolves on one (or multiple) electronic states. In recent years, several contributions have been made to understand the excited state evolution pathway during the photoisomerization [17, 18, 19, 20, 21, 22, 23, 24], however, in this work emerges that the choice of the level of theory (DFT kernel, basis set, solvent description) to be used in the description of the excited state electronic layout is critical to have a correct description of the photophysics of the system. The presented results can be an important starting point to perform subsequent excited state *ab initio* simulations, such as molecular dynamics, for the initial characterization of the FC region, a required preliminary step for the interpretation of several available time‐resolved experiments [17, 18, 21, 22, 23, 79, 80] and for a deeper understanding of the molecular mechanisms underlining the photorelaxation towards an improved design of molecular rotors.

## 2 Methods and Computational Detail

Minimum energy structures were obtained by performing geometry optimization procedures carried out using methods relying on a full quantum mechanical approach. Ground‐ and excited‐state energies, gradients, and higher order properties were obtained by solving the Kohn–Sham equation within DFT and time‐dependent density functional theory (TD‐DFT), respectively [28, 29, 30]. The minimum energy reference structure for the ground state stable isomer was obtained by performing a geometry optimization starting from a geometrical guess resembling the Min1, with the final optimized values for the C
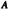
C
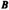
C
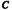
C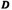 dihedral (see Figure 1 for the labeling scheme) of $-14.35^{\circ }$ and using the hybrid Becke three‐parameter Lee–Yang–Parr (B3LYP) density functional with 6‐31G(d,p) basis set in cyclohexane. Solvent effects were taken into account implicitly by the adoption of the conductor‐like version of the polarizable continuum model [81, 82, 83] (C‐PCM with ϵ=2.0165). Such an optimized structure was retained for all subsequent calculations to individually evaluate the effects on both vertical excitation energies and oscillator strengths of S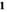 ← S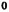 and S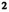 ← S
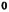
excited state transitions independently from indirect effects of the theory levels on the geometry relaxation. Given the different character of the low‐lying electronic transitions (localized or partially charge transfer), the opportunity of using a range‐separated hybrid functional was evaluated first. A comparison between B3LYP and its range‐separated version with the Coulomb‐Attenuating approach (CAM‐B3LYP) [84] was performed indeed, since by tuning the Hartree–Fock (HF) exchange fraction from 19% to 65% from short‐ to long‐range interelectronic separation, it usually better describes CT transitions than its global‐hybrid counterpart [85, 86].

The necessity for using basis sets with larger spatial extent (i.e., diffuse functions) was evaluated next by using the 6‐31++G(d,p) basis set, since the diffuse functions can be potentially more appropriate to describe the electronic density in excited states, which can be more delocalized in the space [87, 88]. Solvent effects on the electronic transitions by using the non‐equilibrium Linear Response‐C‐PCM formalism and comparing the results with the gas‐phase ones were evaluated next, to better reproduce the experimental conditions. B3LYP versus CAM‐B3LYP kernels, 6‐31G(d,p) versus 6‐31++G(d,p) basis sets, and gas‐phase versus solvent, gave a total of eight combinations for the theory levels for each of the two transitions. Additionally, to accurately describe with DFT non‐covalent interactions, a study was performed in the ground state on the two ground state structures (see Result section) evaluating the opportunity of employing the D3 version of Grimme's dispersion with the original D3 damping function (GD3 correction) [89, 90, 91], See the results section for further detail. Finally, to better evaluate the effect of the implicit solvent on predicting the vertical transition energies, for both the S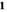 ← S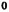 and S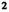 ← S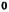 transitions, calculations were performed using two different formalisms for implicit solvation and excited states, which are either the Linear Response (LR) formulation [30, 73, 74], default procedure that computes the solvent effect on the SCF density and then applies the response theory or the so‐called State Specific (SS) [75, 76, 77, 78] approach where a more expensive external iteration procedure is applied to compute the energy in solution by making the solvent reaction field self‐consistent with respect to the solute CI‐like density.

Then, two different theory levels were chosen to be the best compromise between accuracy and computational cost (please see results for a detailed discussion) to perform future, more expensive excited state calculations, that is, *ab initio* molecular dynamics: TD‐CAM‐B3LYP/6‐31++G(d,p)/LR‐C‐PCM and B3LYP/6‐31G(d,p)/C‐PCM in the excited and ground states, respectively. We analyzed the effects of these two different theory levels on the curvature of the potential, and for this aim we computed and compared their Raman spectra in terms of their Raman shift values. Harmonic frequencies and Raman intensities, calculated starting from the same optimized geometry at the B3LYP/6‐31G(d,p)/C‐PCM level of theory using both B3LYP/6‐31G(d,p)/C‐PCM and CAM‐B3LYP/6‐31++G(d,p)/C‐PCM were computed, indeed. All calculations were performed using the electronic structures suite of programs Gaussian 16 [92]. Low‐lying electronic transitions in the Franck–Condon region have also been computed within Multi‐Configurational Self‐Consistent Field (MCSCF) [93, 94, 95, 96, 97] electronic structure methods. In particular, the reference electronic wavefunction has been obtained by Complete Active Space Self Consistent Field (CASSCF) [98, 99] calculations considering 6 electrons in 6 orbitals, using the 6‐31G(d,p) basis set. The State‐Average (SA) considered 8 roots with equal weight (from S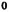 to S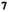), resulting in a SA(8)‐CASSCF(6,6)/6‐31G(d,p) (the active space is reported in Figure S1 in Supporting Information). On top of all SA‐CASSCF calculations, multi‐configurational second‐order perturbation theory (CASPT2) [100, 101, 102, 103, 104] single point energy calculations were performed to account for the necessary dynamic electron correlation employing both single state and multistate (MS) approaches. Additional details are provided in ESI. All CASSCF and CASPT2 calculations were performed with the OpenMolcas (v. 23.10) [105] suite of programs.

## 3 Results and Discussion

### 3.1 Ground State Conformations and Low‐Lying Excited States in the Franck–Condon Region

Understanding the energy separation and the nature of electronic states around the Franck–Condon (FC) region is crucial for the subsequent characterization of the kinetics and mechanism of the photophysics of these systems. For such a purpose, it is not sufficient to control the theory‐level accuracy of just one electronic transition against the experimental value. Instead, it is more fundamental to gauge how different effects may impact the energy separation among the low‐lying electronic states in the FC region and potentially be involved in the photodynamics. To better theoretically study the open issue about the photorelaxation process, we recall here that one (or multiple) low‐lying electronic transition(s) might be experimentally responsible for the first peak in the absorption spectrum in the visible range at ∼3.20 eV (387 nm) [18].

**FIGURE 2:**
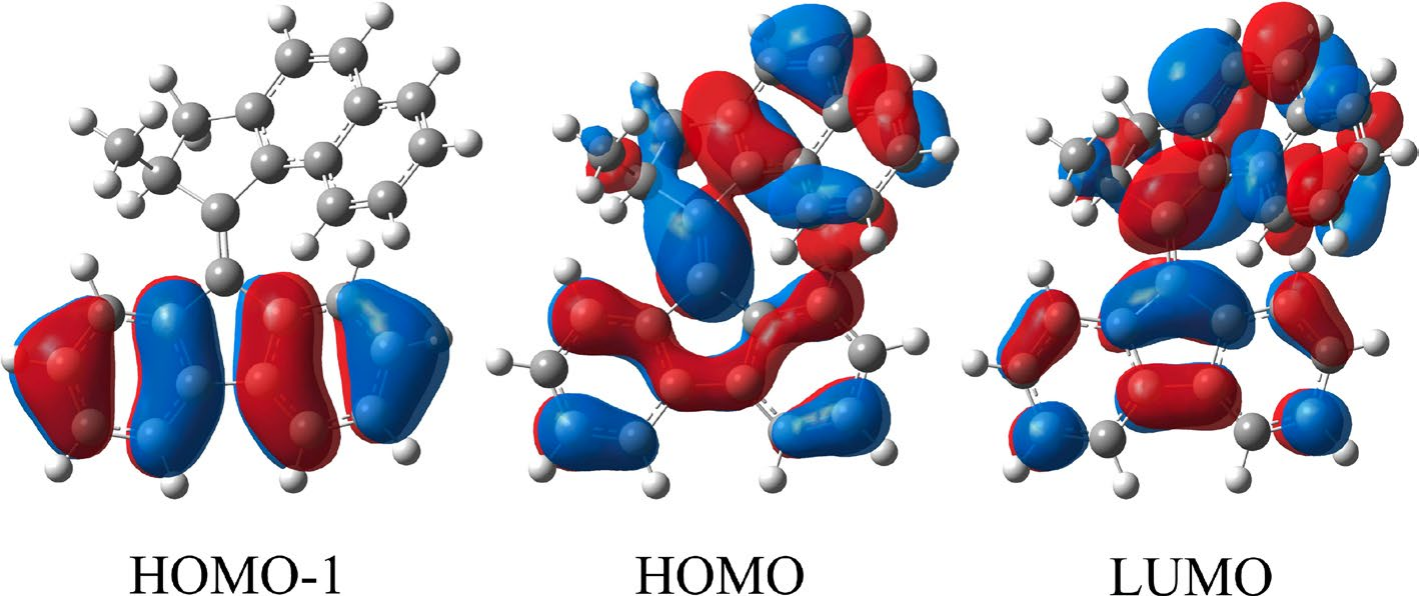
CAM‐B3LYP/6‐31++G(d,p)/C‐PCM cyclohexane canonical frontier molecular orbital isosurfaces (isovalue 0.02). From the left, HOMO‐1, HOMO, and LUMO.

We report in Table 1 the electronic layout of the two low‐lying electronic transitions and their characterization at high level of theory (MS‐CASPT2). This analysis is the starting point for the subsequent description of the system at a more cost‐effective level, such as TD‐DFT. In this region, we found both the S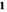 ← S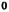 and the S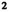 ← S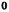 electronic excitations, and they can be described mostly by a HOMO to LUMO, with an high oscillator strength, and by HOMO‐1 to LUMO transition, mostly forbidden, respectively (see Figures 2 and S1 in Supporting Information for molecular orbital spatial representations).

**TABLE 1:** MS‐CASPT2//SA(8)CASSCF(6,6)/6‐31G(d,p) low‐lying excited characterization in gas‐phase. VEEs in eV, oscillator strengths (f, dimensionless), and χ (the main MO contribution to the transitions) are reported. All values were obtained using the Min1 and Min2 optimized geometries at B3LYP/6‐31G(d,p)/C‐PCM in cyclohexane.

	VEE	*f*	χ
Min1		
S 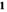 ←S 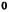	3.49	0.77	HOMO‐LUMO
S 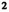 ←S 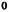	3.72	0.01	HOMO‐1‐LUMO
Min2			
S 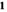 ←S 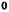	3.11	0.58	HOMO‐LUMO
S 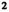 ←S 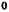	3.55	0.00	HOMO‐1‐LUMO

It is fundamental to rely on a very accurate description of both the two low‐lying S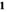 and S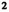 excited states potentially involved in the photophysics and photorelaxation. Thus, we focused on analyzing several theory levels to simultaneously reproduce the two lowest energy transitions in a reliable way. For this aim, it is crucial to also describe the representative conformation(s) of the systems in their ground state equilibrium and the nature of the involved electronic transitions. There have been found two minima energy structures representative of the ground state and involved in the rotatory process discussed in the introduction, corresponding to the two different stable and unstable isomers (Min1 and Min2, respectively; see Figure 1) that differ by 3.54 kcal/mol in energy at B3LYP/6‐31G(d,p)/C‐PCM in cyclohexane. Their main structural difference is the relative orientation of the rotor and stator, represented by the value of the dihedral angle C
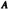
C
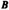
C
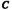
C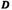, that changes from $-14.35^{\circ }$ for Min1 to $-153.29^{\circ }$ for Min2. The electronic transitions have the same character for both minimum energy structures, where the less stable Min2 shows red‐shifted values for both transitions with respect to the most stable Min1; see values in Table 1. Thus, we mostly focused on the photophysics of the stable isomer, since it is the starting point for the photorelaxation and rotatory cycle in the FC region. The S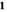 ← S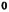 electronic excitation is ascribed to the HOMO to LUMO transition (see Figure 2). Such a transition is a spatially localized excitation on both the fluorene and naphthalene moieties, in which it is possible to observe a reorganization of the electronic density mostly on the C
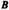
C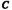 bond. The LUMO has, indeed, a lower bonding character around this region (as witnessed by the depletion of the density, see Figure 2) and thus this excitation might promote the elongation of the bond, leading to the photoisomerization. Concerning the S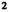 ← S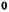 excitation, we observe that it is a HOMO‐1 to LUMO transition with a strong charge transfer (CT) character, and for this reason it can be appropriate to use a potential that can better describe this type of transitions. It is important to note that in the framework of the TD‐DFT, the nature of the electronic excited states needs a careful attention, especially in the case of CT transitions, since the energy order and separation might strongly depend on the choice of the exchange‐correlation functional [85, 86]. We wish also to stress here that also post Hartree–Fock methods, as CASSCF (see Table S1 in Supporting Information), can incur an inaccurate energy order if no further electronic dynamical correlation (PT2) is included. Thus, in the next paragraph we evaluate the effect of the theory level on the accuracy of the description of the energy separation in the FC region, between the lowest excited states relative to the ground state energy minimum structure. This is a very important element to be considered in choosing the accurate potential and to ensure the possibility of studying the photophysics and photorelaxation of these systems, that is, via *ab initio* molecular dynamics in the excited state.

### 3.2 Gauging the Theory Level

Range separated global hybrid DFT functionals, that is, the Coulomb‐Attenuated Method (CAM)‐B3LYP, have been shown to usually outperform global hybrid functionals, that is, B3LYP, for accurately describing the energy layout of excited states when CT excitations are involved [85, 86, 106, 107, 108]. Additionally, it is important to check the balanced effects of the diffuse functions, the solvent and the DFT kernel can have together. Thus, we reported in the Table 2 the computed vertical excitation energies (VEE) and oscillator strengths for both the two low‐lying S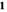 ← S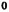 and S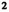 ← S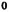 transitions on the Min1 structure and using different levels of theory (see Methods for further detail), along with the resulting energy separation (Δ S
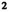
‐S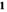).

**TABLE 2:** Vertical excitation energies in eV and oscillator strengths (*f*, dimensionless and in parenthesis) of the S ← S and S ← S electronic transitions, computed at different TD‐DFT levels of theory. Cyclohexane VEEs are obtained using LR‐C‐PCM formalism. The corresponding energy separation (Δ S‐S, in eV) between S and S is also reported in the last column. All values were obtained using the Min1 optimized geometry at B3LYP/6‐31G(d,p)/C‐PCM in cyclohexane.

Level of theory	S 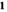 ← S 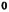	S 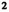 ← S 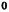	Δ S 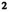 ‐S 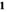
Gas phase			
B3LYP/6‐31G(d,p)	3.11 (0.42)	3.11 (0.04)	0.00
B3LYP/6‐31++G(d,p)	3.05 (0.45)	3.09 (0.00)	0.04
CAM‐B3LYP/6‐31G(d,p)	3.46 (0.55)	3.73 (0.01)	0.28
CAM‐B3LYP/6‐31++G(d,p)	3.39 (0.55)	3.70 (0.01)	0.31
Cyclohexane			
B3LYP/6‐31G(d,p)/C‐PCM	3.02 (0.60)	3.15 (0.00)	0.13
B3LYP/6‐31++G(d,p)/C‐PCM	2.97 (0.61)	3.13 (0.00)	0.17
CAM‐B3LYP/6‐31G(d,p)/C‐PCM	3.35 (0.71)	3.77 (0.01)	0.41
CAM‐B3LYP/6‐31++G(d,p)/C‐PCM	3.29 (0.72)	3.74 (0.01)	0.44

We first analyzed the gas‐phase TD‐DFT results by comparing them with the excitation energies obtained using the reference theory level, that is, MS‐CASPT2 (see Table 1). TD‐CAM‐B3LYP gas phase results are the ones that resemble the most the reference calculations, given the noticeable blue shift with respect to the analogous TD‐B3LYP vertical excitation energies. It is worth noticing that moving from B3LYP to CAM‐B3LYP, a large shift of ∼ +0.35 eV and of ∼ +0.6 eV is observed for the S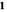 ← S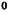 and S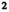 ← S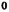 transitions, respectively. It is also important to note here that if we compare CAM‐B3LYP with B3LYP results (in gas‐phase and using 6‐31G(d,p) ) with the analogous calculation at MS‐CASPT2 level (see the first two rows of Table 1 and the third row of Table 2), there is a very excellent agreement between TD‐CAM‐B3LYP and MS‐CASPT2 method (3.46 vs. 3.49 and 3.73 vs. 3.72 eV), while B3LYP erroneously describes such transitions as degenerate and CASSCF, with no further PT2 correction, wrongly assigns their energy order (see Table S1 in Supporting Information). Additionally, TD‐B3LYP excited states show a very small energy separation in gas phase (almost degenerate), while TD‐CAM‐B3LYP can accurately separate S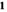 and S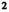 as seen in the reference theory level, even if there is a slight overestimation of the energy separation (0.27 eV vs. 0.23 eV). To have a better and comprehensive description of the PES around the Franck–Condon region, we also checked such energy separation and agreement using a geometry that is distorted along the two main coordinates involved in the photoisomerizaion, the dihedral angle C
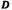
C
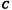
C
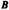
C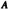 related to the torsion of the rotor with respect to the stator and the dihedral angle C
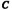
C
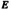
C
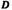
C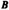 related to the pyramidalization of the carbon C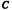. Such distortions are obtained starting from the Min1 and following the geometrical distortion observed in References [20] and [15] (see Figure S2 in Supporting Information for details on the structure and its justification). On this distorted geometry, such energy separation increases at ∼0.6 eV (see Table S2 in Supporting Information), in good agreement with MS‐CASPT2 (∼0.7 eV), see Table S2 in Supporting Information. Finally, if we perform the same analysis on the Min2, we still observe an optimal accordance in the energy separation, where TD‐CAM‐B3LYP/6‐31G(d,p) reproduces the MS‐CASPT2 in gas phase (0.45 eV, see Table S2 in Supporting Information vs. 0.44 eV, see Table 1).

We now consider the introduction of diffuse functions, as these ones can be more appropriate to describe the electronic density in the excited states, which can be more delocalized (diffused) in the space. So, from the inspection of Table 2 by including the diffuse functions, moving from the odd to even rows, we observe a consistent red‐shift of VEEs of ∼ −0.06 eV and ∼ −0.03 eV for the S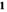 ← S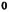 and S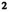 ← S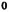 transitions, respectively. Thus, the introduction of diffuse functions: (i) improves the accuracy of CAM results (and worsens B3LYP ones, instead) and (ii) increases the separation between the two excited states (+0.03 eV increased separation), as it can be seen by the analysis of the values in the last column of Table 2. Finally, the introduction of the solvation reaction field, via an implicit model in the LR regime, results in a red‐shift of ∼ −0.10 eV and an opposite blue‐shift of ∼ +0.04 eV for the S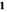 ← S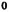 and S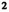 ← S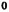 transitions, respectively. Thus, the solvent increases by +0.14 eV the energy separation between the two states. Summing up all the effects, we see that moving from B3LYP to CAM‐B3LYP, from 6‐31G(d,p) to 6‐31++G(d,p), and from gas to C‐PCM a total of +0.44 eV (0.27+0.03+0.14 eV) energy separation is obtained between the two excited states (see last row and column of Table 2). Then, we analyze the oscillator strengths, and we observe for the S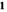 ← S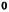 excitation a progressive increase moving from gas to solvent (∼ +0.15) and from B3LYP to CAM‐B3LYP (∼ +0.12). A very small increment, sometimes negligible, has the introduction of the diffuse functions on the oscillator strength, instead. We see that these effects are mostly additive, and the first electronic transition becomes 70% brighter moving from B3LYP/6‐31G(d,p) to CAM‐B3LYP/6‐31++G(d,p)/C‐PCM. No significant changes in the oscillator strength of the S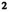 ← S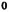 are observed, where all theory levels here analyzed indicate the S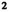 ← S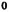 as a dark transition.

**FIGURE 3:**
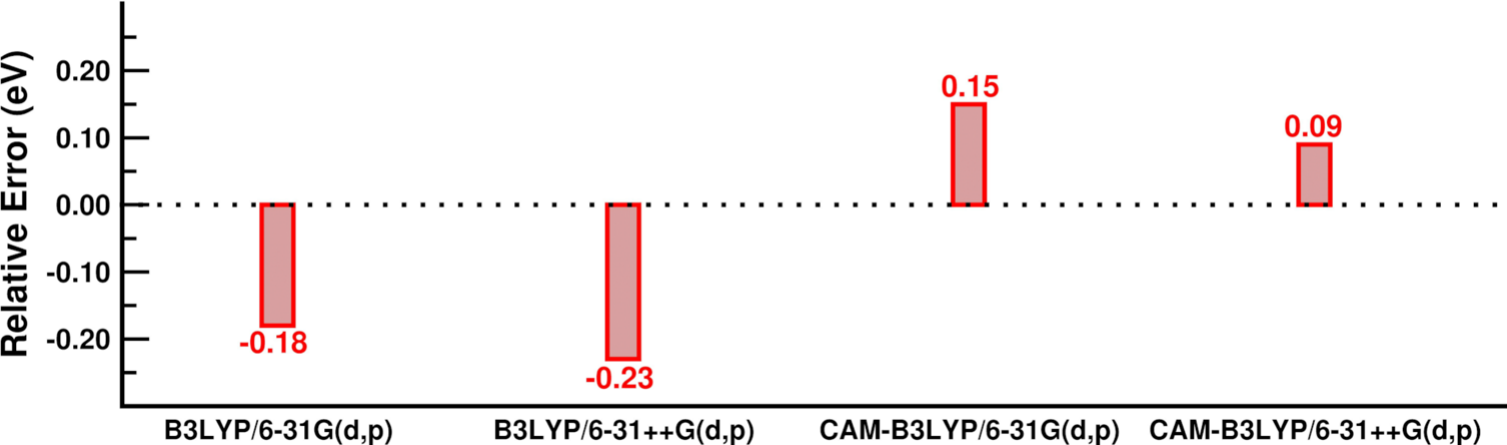
Relative error analysis. Values are reported in eV for the different theory levels in C‐PCM and are computed as the difference between the computed VEE for the S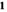 ← S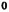 (the bright transition for all cases, see Table 2) and the experimental value of the maximum absorption, 3.20 eV (387 nm) [18].

We finally reported in the Figure 3 the accuracy of the different theory levels in solution, computed as the difference between the computed VEEs for the S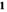 ← S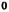 (the bright transitions for all cases, reported in the second half of Table 2) and the experimental value of the maximum absorption in the same condition. It is worth noting how the B3LYP shows the largest errors (∼ −0.2 eV), while TD‐CAM‐B3LYP/6‐31++G(d,p)/C‐PCM provides the best match also with the experiment (in addition to be, CAM‐B3LYP, the best match in the same conditions with the reference level as shown before), with a blue shift of less than 0.1 eV. Although, it is important to recall here that the VEEs cannot be directly compared with the maximum of the experimental absorption due to the high‐order effects (i.e., vibronic progression, asymmetric broadening, etc.) [109, 110].

Thus, TD‐CAM‐B3LYP/6‐31++G(d,p)/C‐PCM is in our opinion, the most reliable choice for describing the excited state electronic layout in cyclohexane since it takes into account all the effects (solvent, diffuse density, tuned HF exchange) and shows one of the lowest errors. From this analysis, is also clear that CAM‐B3LYP/6‐31++G(d,p)/C‐PCM describes a significant energetic separation between the S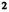 and S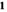 electronic states (+0.44 eV), while B3LYP/6‐31G(d,p) shows them as degenerate instead. This is also observed for the Min2, which at CAM‐B3LYP/6‐31++G(d,p)/C‐PCM exhibits VEEs of 2.93 (S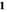 ← S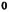, osc. strength of 0.64) and 3.55 (S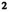 ← S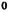, osc. strength of 0.01) eV, with the same character of two low‐lying excitations observed in Min1 and described before.

In conclusion, we recommend CAM‐B3LYP/6‐31++G(d,p)/C‐PCM as a level of theory to adopt for the calculation of excited state properties, that is, electronic excited state dynamics. This is motivated by the presence of the CAM‐B3LYP functional, as reported previously is the recommended functional for describing CT electronic excitations by the presence of a more complete basis set, with the introduction of the diffuse functions, that increases the accuracy of the results and by the presence of the solvent to reproduce the experimental conditions. Moreover, we believe that, among the level of theory showed that CAM‐B3LYP/6‐31++G(d,p)/C‐PCM can also better describe simultaneously the S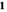 and S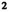 excited states and therefore the energy separation, which reaches the maximum precisely at this potential. While B3LYP artificially describes a close degeneracy of the two states. The last aspect is important to be able to perform an accurate *ab initio* dynamics of the excited state, like the Born‐Oppenheimer dynamics on this molecule. Once we have chosen this theory level for the photophysics of the system, we analyze in the next paragraphs the individual effects of accurately describing non‐covalent interactions and state‐specific solvation effects.

### 3.3 Effect of the Empirical Dispersion

**FIGURE 4:**
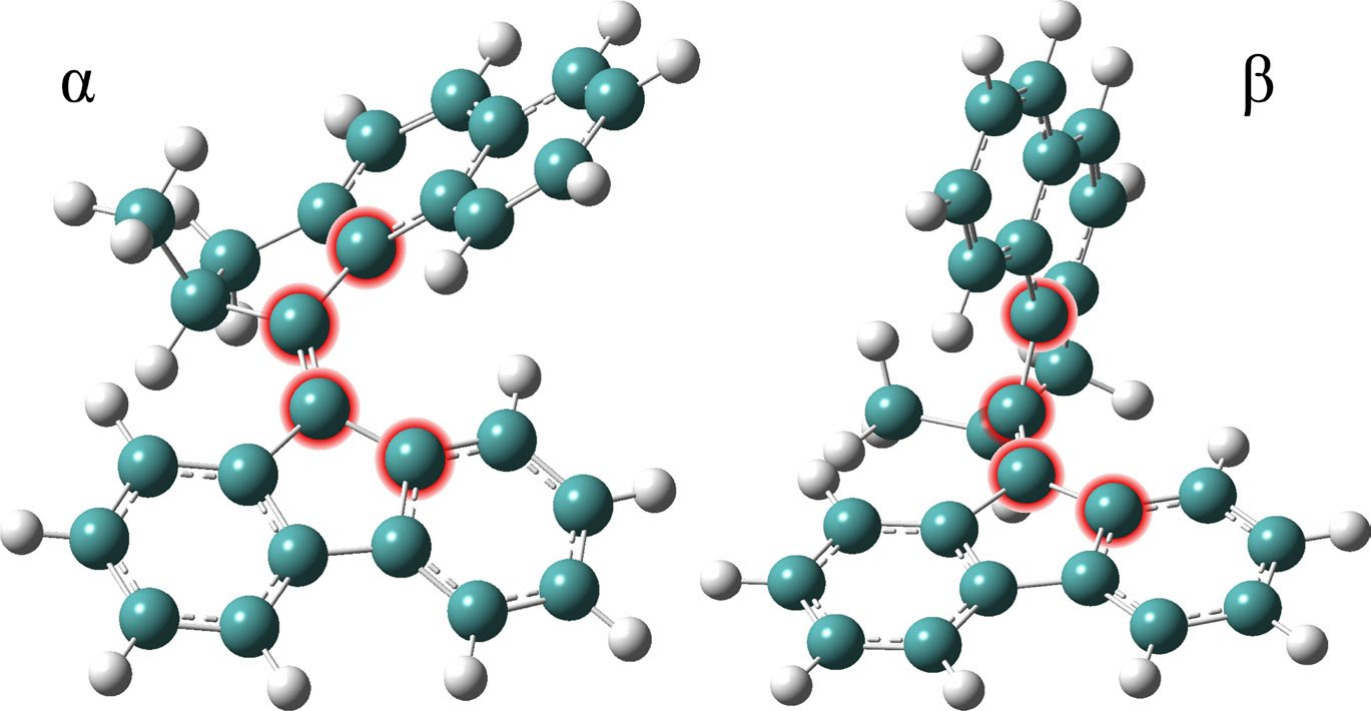
CAM‐B3LYP/6‐31++G(d,p) minimum energy structure (left, namely α) and the corresponding distorted structure along the red highlighted dihedral angle (right, namely β). See text for detail.

As shown in the previous paragraph, we consider the CAM‐B3LYP/6‐31++G(d,p)/C‐PCM cyclohexane, the best compromise for the study of the excited state photorelaxation and photophysics, that is, to be employed in excited state *ab initio* molecular dynamics simulations. To evaluate how such a theory level is capable of accurately describing non‐covalent interactions (i.e., among the aromatic and/or methyl portions of the system) present in the molecule, we tested the inclusion of dispersion effects, evaluating the opportunity of employing Grimme's dispersion with the original D3 damping function (GD3 correction) [89, 90, 91]. For this aim, we chose two structures that differ the most in the relative orientation of the fluorene and naphthalene portions. We started from the minimum energy geometry (stable isomer) optimized at CAM‐B3LYP/6‐31++G(d,p) theory level, and we denoted this geometry as α. A second geometry, denoted as β, was obtained from the first, varying the C
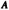
C
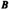
C
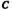
C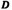 dihedral angle until we have nearly perpendicular orientation of the rotor with respect to the stator (see Figure 4). More specifically, the value of this dihedral angle for the α geometry corresponds to $-13.41^{\circ }$, while the same parameter for the β geometry corresponds to $-66.00^{\circ }$. The α and β geometries were chosen to analyze non‐covalent interactions in two limit structures, neglecting or including the dispersion contribution to the energy and how this can have a different weight on the energy difference and stability of these conformations. Using these two conformations, we reported in Table 3 the effect of including or not the empirical dispersion (GD3), using two different levels of theory, B3LYP/6‐31G(d,p) and CAM‐B3LYP/6‐31++G(d,p), both in gas phase, without the inclusion of the solvent effects and further geometry optimization, since our main aim here was to only monitor the GD3 correction on the potential.

**TABLE 3:** Energy difference between the two geometries shown in the Figure 4, at different levels of theory. ΔE (kcal/mol) refers to E ‐ E. Computed as single energy point calculations on the α and β geometries with no further geometry optimization procedure.

	ΔE NO GD3	ΔE GD3
B3LYP/6‐31G(d,p)	35.86	37.88
CAM‐B3LYP/6‐31++G(d,p)	39.34	40.81

From this analysis it is clear that we can consider the effect of the empirical dispersion very small and negligible. The introduction of GD3 in the B3LYP/6‐31G(d,p) potential leads to an increase in the energy difference between the two geometries of only ∼2 kcal/mol, that becomes even smaller in CAM‐B3LYP/6‐31++G(d,p), an increase of ∼1.5 kcal/mol. We are therefore confident in suggesting that the inclusion of GD3 dispersion is not mandatory in the description of the rotatory process.

### 3.4 The Treatment of the Solvent Effects

We consider the importance of which approach is the most appropriate to describe the solvent effects on the electronic excitations in this system. Usually, explicit solvent description is preferred, although it can be very time‐consuming, so here we gauged the accuracy of cost‐effective implicit solvation models, fundamental to obtain accurate results in reasonable time for excited state simulations. In these regards, implicit solvent models need to be tested in different response formalisms, to be sure that the environment response is accurately described when the electronic density changes upon the photoexcitation. Thus, we computed excitation energies in solution using two different formalisms for implicit solvation, that are either the LR formulation or the SS approach (see Methods for a detailed discussion). While the first approach is much more convenient computationally [30, 73, 74], the second can better account for the polarization of the solvent in the presence of the excited electronic density of the solute, that is, for the difference of the dipole moment between the ground and the excited state. Therefore, the SS approach is in principle more accurate, although the overall result (the VEEs in solvent) also depends on the combination with the density functional accuracy. To gauge the accuracy of the TD‐DFT/C‐PCM combined potential and to perform the best choice for our study, we tested the performance of the B3LYP and the CAM‐B3LYP density functionals with both the LR and SS C‐PCM formalisms starting from the optimized geometry of the stable isomer computed at B3LYP/6‐31G(d,p)/C‐PCM cyclohexane level of theory. We calculated with both approaches the S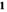 ← S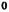 and S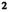 ← S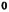 VEEs for the molecule in cyclohexane. We used the 6‐31++G(d,p) basis set in all cases and the optimized geometry of the stable isomer. Results are collected in the Table 4, along with the results obtained in gas‐phase for a better comparison.

**TABLE 4:** LR‐ versus SS‐C‐PCM TD‐DFT VEEs in eV of the S ← S and S ← S electronic transitions. All values were obtained using the Min1 optimized geometry at B3LYP/6‐31G(d,p)/C‐PCM in cyclohexane. Gas‐phase values are reported as well for a better comparison. The corresponding energy separation (Δ S‐S, in eV) between S and S is also reported in the last column.

B3LYP/6‐31++G(d,p)	S 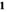 ← S 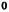	S 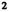 ← S 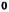	Δ S 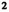 ‐S 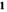
Gas phase	3.05	3.09	0.04
Linear response	2.97	3.13	0.17
State specific	3.06	2.90	‐0.15
CAM‐B3LYP/6‐31++G(d,p)	S 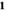 ← S 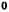	S 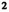 ← S 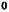	Δ S 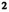 ‐S 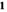
Gas phase	3.39	3.70	0.31
Linear response	3.29	3.74	0.44
State specific	3.39	3.58	0.19

For what regards the S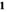 transition, we observe from Table 4 a red‐shift of about ∼ −0.1 eV induced by the solvent when treated by the LR formalism. On another hand, the solvent effect is practically nihil when adopting the SS formalism, obtained back the result in gas‐phase. This last result is not surprising, being the S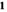 ← S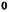 locally excited in nature, and reasonably characterized by a small difference between the dipole moment in S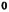 and S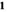 [75, 76, 77, 78, 111]. For what regards the S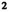 transition, we observe from Table 4 a red‐shift of −0.19 or −0.12 eV induced by the solvent when treated by the SS formalism for the B3LYP and CAM‐B3LYP results, respectively. On the contrary, the LR solvent effect is a blue‐shift of ∼ +0.04 eV in both B3LYP and CAM‐B3LYP VEEs. Having the S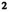 ← S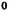 transition a charge transfer nature, it is reasonably characterized by a significant change of the dipole upon excitation with respect to S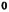. Therefore, we can consider the SS approach more sensitive to this change, taking into account for this difference in the solute‐solvent interaction in the CI‐density.

Hence, we can consider the LR approach more appropriate in the description of the solvent effect for the description of S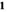 state, while the S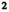 is described more accurately by the SS formalism. Therefore, S
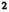
(CAM‐B3LYP/SS‐C‐PCM)‐S
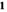
(CAM‐B3LYP/LR‐C‐PCM) is the best estimate of the S
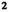
‐S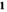 difference, that is, about ∼0.3 eV. However, by inspection of Table 4, we observe that the CAM‐B3LYP/LR approach gives the best approximation of this result (0.44 eV). In summary, the CAM‐B3LYP/6‐31++G(d,p)/LR‐C‐PCM is the potential that can deliver the best accuracy for the simultaneous description of both excited states considered in this work and the overall electronic layout of the low‐lying excited state. In addition to that, LR‐C‐PCM is both less computationally expensive than SS‐C‐PCM and usually has well‐established nuclear gradient expressions already implemented in most of the electronic structure codes for performing more involved excited state calculations, that is, *ab initio* MD and geometry optimizations in the excited states.

### 3.5 DFT Kernel Effects on Potential Curvature

**FIGURE 5:**
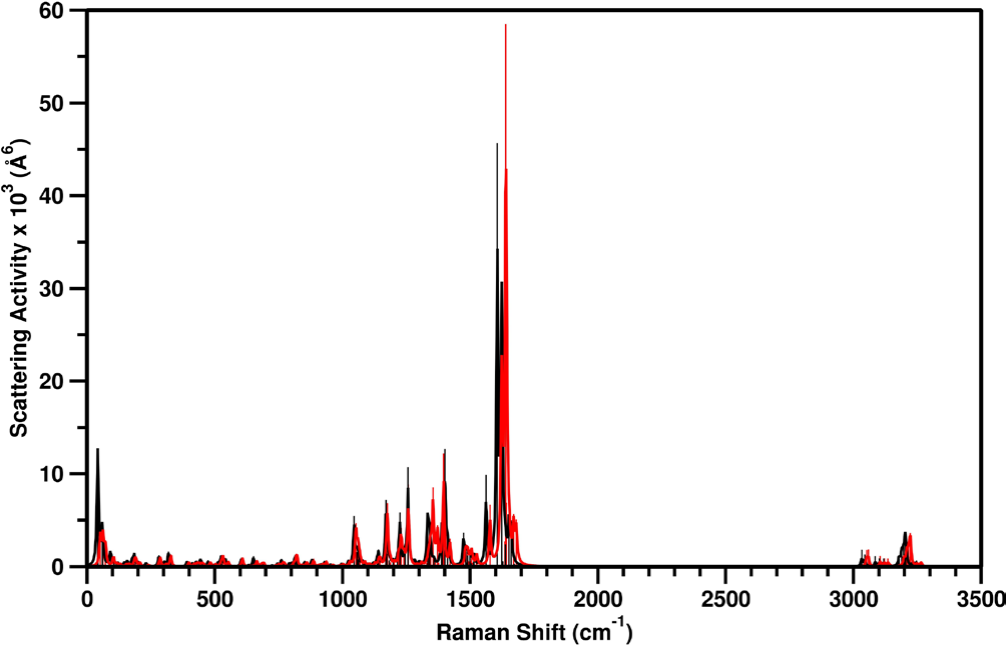
Raman spectra: B3LYP/6‐31G(d,p)/C‐PCM cyclohexane (in black) and CAM‐B3LYP/6‐31++G(d,p)/C‐PCM cyclohexane (in red). B3LYP/6‐31G(d,p)/C‐PCM cyclohexane optimized geometry is used in both cases.

We recall here the fact that the electronic ground state can be accurately described by a cheaper but still accurate theory level such as B3LYP/6‐31G(d,p)/C‐PCM, while for the detailed description of excited states we suggest using CAM‐B3LYP/6‐31++G(d,p)/LR‐C‐PCM. Thus, in this paragraph we analyze the effects of these two different theory levels on the curvature of the potential (to perform, i.e., molecular dynamics or geometry optimizations). For this aim we computed and analyzed the Raman spectra in terms of their Raman shift values. Such analysis can ensure the consistency on the excited states force constants of the introduction of both the range separated hybrid functional and the diffuse functions, required to better describe the nature of the excited states. Moreover, by this comparison, we can understand if there might be any change in the potential curvature and resulting forces if the theory level is changed from B3LYP/6‐31G(d,p)/C‐PCM to CAM‐B3LYP/6‐31++G(d,p)/C‐PCM (i.e., for excited state *ab initio* MD). We computed indeed the Raman spectra for both theory levels, and the results are reported in Figure 5, and a quantitative analysis of such differences on selected Raman active modes is summarized in Table 5. These selected Raman active modes involve, in different combinations, the C=C stretchings of naphthalene and/or fluorene, the C
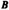
=C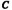 stretching, and several C=C‐H scissoring and H‐C‐H wagging modes (see Figure S3 in ESI). The spectra look very similar both in the Raman activity and shift, although we note a very limited blue shift of the Raman shift for the CAM‐B3LYP/6‐31++G(d,p)/C‐PCM compared to B3LYP/6‐31G(d,p)/C‐PCM and a not negligible change in the Raman activity (not connected to the potential curvature) in the main peak around ∼1600 (see Table 5).

**TABLE 5:** Selected active Raman mode comparison (see Figure 5 for the full spectra). Frequencies are in cm and Raman activity in ${\text{Å}}^{4}$/AMU. B3LYP/6‐31G(d,p)/C‐PCM cyclohexane values, along with the difference between the B3LYP/6‐31G(d,p)/C‐PCM cyclohexane and CAM‐B3LYP/6‐31++G(d,p)/C‐PCM cyclohexane levels of theory, are reported in the second and last columns, respectively. B3LYP/6‐31G(d,p)/C‐PCM cyclohexane optimized geometry is used in both cases. Vector representation of the selected Raman active normal modes (**A**, **B**, **C**, **D**, and **E**) is reported in Figure S3 in ESI.

Normal mode	Frequency	Δ Frequency
	(Raman activity)	(Δ Raman activity)
**A**: twisting of C=C‐H in fluorene+	303 (9)	−7 (4)
partial pyramidalization C 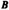 carbon		
**B**: alternating stretching of naphthalene C=C bonds+	1401 (1048)	5 (45)
partial scissoring of C=C‐H in naphthalene+		
partial wagging of the H‐C‐H in the cyclopentene moiety		
**C**: scissoring of C=C‐H in naphthalene+	1562 (911)	−16 (291)
partial stretching of naphthalene C=C bonds		
**D**: alternating stretching of naphthalene C=C bonds+	1606 (4343)	−17 (1616)
partial C 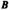 =C 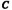 stretching		
**E**: C 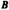 =C 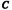 stretching+	1623 (2507)	−17 (−3179)
alternating stretching of naphthalene and fluorene C=C bonds		

From this analysis, we are confident that despite the small differences, all analyzed modes undergo a limited shifts moving towards the CAM‐B3LYP potential, and the mode composition is also very similar. The change of the intensity does not significantly affect the potential curvature; indeed, the main results here are that the variations in the Raman shift that are linked to the force constants, are negligible, ensuring the possibility to collect ground state dynamics with B3LYP/6‐31G(d,p)/C‐PCM and move directly to excited state MD using CAM‐B3LYP/6‐31++G(d,p)/C‐PCM.

## 4 Conclusions

In this study we propose a detailed analysis of the electronic layout of a second‐generation LDMRM, as a preliminary mandatory step for ensuring an accurate description of the early stage of the photoexcitation process in solution of such a class of compounds. Although we present a detailed analysis focused on a molecular rotor belonging to the second generation, we think that the nature of the low‐lying electronic states here found (localized on both or one half of the system versus charge transfer states) is quite common in molecular rotors belonging to the overcrowded alkene class. We began with the characterization of the minimum energy structure(s) representative of the equilibrium and the nature of the excited states in the FC region. Then, we tested different TD‐DFT levels of theory for the description of the electronic layout of the low‐lying excited states, presenting also a careful comparison employing refined methods such as MS‐CASPT2. The combined effects of the DFT kernel (global‐hybrid vs. range‐separated hybrid), basis set extension, empirical dispersion, and solvation response approaches were analyzed, showing how they can lead to both similar errors with respect to the experimental absorption but also to a completely different description of the electronic layout, potentially predicting wrong photophysics and kinetics if not carefully investigated. Since it is crucial to have an accurate description of the environmental effects we carefully compared SS‐TDDFT results with LR‐TDDFT ones, ensuring that CAM‐B3LYP/6‐31++G(d,p)/LR‐C‐PCM is the potential that can deliver the best accuracy for the simultaneous description of both excited states with an affordable computational cost. The inclusion of the empirical dispersion with the GD3 formalism was also shown to be not mandatory for excited state simulations of this system. Our main result is the identification of two cost‐effective theory levels: one for the ground state, using the B3LYP functional with the 6‐31G(d,p) basis set and the conductor‐like polarizable continuum model for solvation, and another for the excited states, employing the Coulomb‐Attenuating Method version (CAM‐B3LYP) functional with the double‐ζ split‐valence basis set with polarization and diffuse functions and the linear response C‐PCM solvation model. Finally we verified how switching between these two different levels of theory can influence the curvature of the potential via a frequency analysis of the normal modes of vibrations active in the Raman spectrum. This work can represent a preparatory step for future computational studies for assessing the required theory levels towards the accurate and cost‐effective treatment of the FC region for similar systems where excited states do not present a double excitation character and solvation effects play important roles. We believe these findings provide a crucial preliminary step for future computations aimed at interpreting a range of time‐resolved spectroscopy experiments.

## Author Contributions

**Raoul Carfora and Federico Coppola:** data curation, investigation, validation, visualization, formal analysis, methodology, writing original draft, writing – review and editing. **Paola Cimino:** validation, investigation, writing – review and editing. **Alessio Petrone and Nadia Rega:** conceptualization, funding acquisition, project administration, supervision, writing – original draft, writing – review and editing.

## Conflicts of Interest

The authors declare no conflicts of interest.

## Supporting information


Supplementary Data S1.

## Data Availability

The data that supports the findings of this study are available in the supplementary material of this article.

## References

[1] V. Balzani , A. Credi , and M. Venturi , Molecular Devices and Machines: Concepts and Perspectives for the Nanoworld (Weinheim, Germany: John Wiley & Sons, 2008).

[2] Y. B. Zheng , Q. Hao , Y.‐W. Yang , B. Kiraly , I.‐K. Chiang , and T. J. Huang , “Light‐Driven Artificial Molecular Machines,” Journal of Nanophotonics 4 (2010): 042501.

[3] C. J. Bruns and J. F. Stoddart , The Nature of the Mechanical Bond: From Molecules to Machines (Hoboken, New Jersey: John Wiley & Sons, 2016).

[4] S. Kassem , T. van Leeuwen , A. S. Lubbe , M. R. Wilson , B. L. Feringa , and D. A. Leigh , “Artificial molecular motors,” Chemical Society Reviews 46 (2017): 2592. 28426052 10.1039/c7cs00245a

[5] M. Baroncini , S. Silvi , and A. Credi , “Photo‐and Redox‐Driven Artificial Molecular Motors,” Chemical Reviews 120 (2019): 200. 31415169 10.1021/acs.chemrev.9b00291

[6] S. Corra , M. Curcio , and A. Credi , “Photoactivated Artificial Molecular Motors,” JACS Au 3 (2023): 1301. 37234111 10.1021/jacsau.3c00089 PMC10207102

[7] A. Singhania , S. Kalita , P. Chettri , and S. Ghosh , “Accounts of Applied Molecular Rotors and Rotary Motors: Recent Advances,” Nanoscale Advances 5 (2023): 3177. 37325522 10.1039/d3na00010a PMC10262963

[8] L. Zhang , H. Wu , X. Li , H. Chen , R. D. Astumian , and J. F. Stoddart , “Artificial Molecular Pumps,” Nature Reviews Methods Primers 4 (2024): 13.

[9] A. Mondal , R. Toyoda , R. Costil , and B. L. Feringa , “Chemically Driven Rotatory Molecular Machines,” Angewandte Chemie, International Edition 61 (2022): e202206631. 35852813 10.1002/anie.202206631 PMC9826306

[10] L. Zhang , Y. Qiu , W.‐G. Liu , et al., “An Electric Molecular Motor,” Nature 613 (2023): 280. 36631649 10.1038/s41586-022-05421-6 PMC9834048

[11] N. Koumura , R. W. Zijlstra , R. A. van Delden , N. Harada , and B. L. Feringa , “Light‐Driven Monodirectional Molecular Rotor,” Nature 401 (1999): 152. 10490022 10.1038/43646

[12] C. Garcia‐Iriepa , M. Marazzi , F. Zapata , A. Valentini , D. Sampedro , and L. M. Frutos , “Chiral Hydrogen Bond Environment Providing Unidirectional Rotation in Photoactive Molecular Motors,” Journal of Physical Chemistry Letters 4 (2013): 1389. 26282290 10.1021/jz302152v

[13] J. Wang , B. Oruganti , and B. Durbeej , “Unidirectional Rotary Motion in Isotopically Chiral Molecular Motors: A Computational Analysis,” Organic Letters 22 (2020): 7113. 32822192 10.1021/acs.orglett.0c02436 PMC7506945

[14] D. R. Pooler , A. S. Lubbe , S. Crespi , and B. L. Feringa , “Designing Light‐Driven Rotary Molecular Motors,” Chemical Science 12 (2021): 14964. 34909140 10.1039/d1sc04781g PMC8612399

[15] P. Roy , A. S. Sardjan , W. R. Browne , B. L. Feringa , and S. R. Meech , “Excited State Dynamics in Unidirectional Photochemical Molecular Motors,” Journal of the American Chemical Society 146 (2024): 12255. 38656968 10.1021/jacs.4c01019 PMC11082934

[16] Y. Li , F. Liu , B. Wang , Q. Su , W. Wang , and K. Morokuma , “Different Conical Intersections Control Nonadiabatic Photochemistry of Fluorene Light‐Driven Molecular Rotary Motor: A Casscf and Spin‐Flip Dft Study,” Journal of Chemical Physics 145 (2016a): 244311. 28049297 10.1063/1.4972825

[17] J. Conyard , K. Addison , I. A. Heisler , et al., “Ultrafast Dynamics in the Power Stroke of a Molecular Rotary Motor,” Nature Chemistry 4 (2012): 547. 10.1038/nchem.1343 22717439

[18] J. Conyard , A. Cnossen , W. R. Browne , B. L. Feringa , and S. R. Meech , “Chemically Optimizing Operational Efficiency of Molecular Rotary Motors,” Journal of the American Chemical Society 136 (2014): 9692. 24918780 10.1021/ja5041368

[19] S. Amirjalayer , A. Cnossen , W. R. Browne , B. L. Feringa , W. J. Buma , and S. Woutersen , “Direct Observation of a Dark State in the Photocycle of a Light‐Driven Molecular Motor,” Journal of Physical Chemistry A 120 (2016): 8606. 27684513 10.1021/acs.jpca.6b09644 PMC5098230

[20] X. Pang , X. Cui , D. Hu , et al., “Watching the Dark State in Ultrafast Nonadiabatic Photoisomerization Process of a Light‐Driven Molecular Rotary Motor,” Journal of Physical Chemistry A 121 (2017): 1240. 28103031 10.1021/acs.jpca.6b12253

[21] C. R. Hall , J. Conyard , I. A. Heisler , et al., “Ultrafast Dynamics in Light‐Driven Molecular Rotary Motors Probed by Femtosecond Stimulated Raman Spectroscopy,” Journal of the American Chemical Society 139 (2017): 7408. 28486804 10.1021/jacs.7b03599

[22] C. R. Hall , W. R. Browne , B. L. Feringa , and S. R. Meech , “Mapping the Excited‐State Potential Energy Surface of a Photomolecular Motor,” Angewandte Chemie, International Edition 57 (2018): 6203. 29633492 10.1002/anie.201802126

[23] P. Roy , A. S. Sardjan , A. Cnossen , W. R. Browne , B. L. Feringa , and S. R. Meech , “Excited State Structure Correlates With Efficient Photoconversion in Unidirectional Motors,” Journal of Physical Chemistry Letters 12 (2021): 3367. 33784091 10.1021/acs.jpclett.1c00710

[24] J. Wen , S. Mai , and L. González , “Excited‐State Dynamics Simulations of a Light‐Driven Molecular Motor in Solution,” Journal of Physical Chemistry A 127 (2023): 9520. 37917883 10.1021/acs.jpca.3c05841 PMC10658450

[25] M. M. Pollard , A. Meetsma , and B. L. Feringa , “A Redesign of Light‐Driven Rotary Molecular Motors,” Organic & Biomolecular Chemistry 6 (2008): 507. 18219421 10.1039/b715652a

[26] A. Kazaryan , J. C. Kistemaker , L. V. Schäfer , W. R. Browne , B. L. Feringa , and M. Filatov , “Understanding the Dynamics Behind the Photoisomerization of a Light‐Driven Fluorene Molecular Rotary Motor,” Journal of Physical Chemistry A 114 (2010): 5058. 20349978 10.1021/jp100609m

[27] H. Lischka , D. Nachtigallova , A. J. Aquino , et al., “Multireference Approaches for Excited States of Molecules,” Chemical Reviews 118 (2018): 7293. 30040389 10.1021/acs.chemrev.8b00244

[28] R. Bauernschmitt and R. Ahlrichs , “Treatment of Electronic Excitations Within the Adiabatic Approximation of Time Dependent Density Functional Theory,” Chemical Physics Letters 256 (1996): 454.

[29] M. E. Casida , C. Jamorski , K. C. Casida , and D. R. Salahub , “Molecular Excitation Energies to High‐Lying Bound States From Time‐Dependent Density‐Functional Response Theory: Characterization and Correction of the Time‐Dependent Local Density Approximation Ionization Threshold,” Journal of Chemical Physics 108 (1998): 4439.

[30] G. Scalmani , M. J. Frisch , B. Mennucci , J. Tomasi , R. Cammi , and V. Barone , “Geometries and Properties of Excited States in the Gas Phase and in Solution: Theory and Application of a Time‐Dependent Density Functional Theory Polarizable Continuum Model,” Journal of Chemical Physics 124 (2006a): 094107. 10.1063/1.2173258 16526845

[31] C. Daniel , Absorption Spectroscopy, Emissive Properties, and Ultrafast Intersystem Crossing Processes in Transition Metal Complexes: TD‐DFT and Spin‐Orbit Coupling (Cham, Switzerland: Springer International Publishing, 2016), 377–413, 10.1007/128_2015_635. 26129697

[32] S. Lee , M. Filatov , S. Lee , and C. H. Choi , “Eliminating Spin‐Contamination of Spin‐Flip Time Dependent Density Functional Theory Within Linear Response Formalism by the Use of Zeroth‐Order Mixed‐Reference (Mr) Reduced Density Matrix,” Journal of Chemical Physics 149 (2018): 104101. 30219009 10.1063/1.5044202

[33] A. A. Granovsky , “Extended Multi‐Configuration Quasi‐Degenerate Perturbation Theory: The New Approach to Multi‐State Multi‐Reference Perturbation Theory,” Journal of Chemical Physics 134 (2011): 214113. 21663350 10.1063/1.3596699

[34] S. Lee , S. Shostak , M. Filatov , and C. H. Choi , “Conical Intersections in Organic Molecules: Benchmarking Mixed‐Reference Spin–Flip Time‐Dependent Dft (Mrsf‐Td‐Dft) vs Spin–Flip Td‐Dft,” Journal of Physical Chemistry A 123 (2019): 6455. 31283235 10.1021/acs.jpca.9b06142

[35] J. Bloino , A. Baiardi , and M. Biczysko , “Aiming at an Accurate Prediction of Vibrational and Electronic Spectra for Medium‐To‐Large Molecules: An Overview,” International Journal of Quantum Chemistry 116 (2016): 1543.

[36] E. Q. Chong , D. B. Lingerfelt , A. Petrone , and X. Li , “Classical or Quantum? A Computational Study of Small Ion Diffusion in Ii‐Vi Semiconductor Quantum Dots,” Journal of Physical Chemistry C 120 (2016): 19434, 10.1021/acs.jpcc.6b05883.

[37] A. Petrone , D. B. Williams‐Young , D. B. Lingerfelt , and X. Li , “Ab Initio Excited State Transient Raman Analysis,” Journal of Physical Chemistry A 121 (2017): 3958, 10.1021/acs.jpca.7b02905. 28467699

[38] M. H. Palmer , M. Biczysko , K. A. Peterson , C. S. Stapleton , and S. P. Wells , “Structural and Vibrational Properties of Iodopentafluorobenzene: A Combined Raman and Infrared Spectral and Theoretical Study,” Journal of Physical Chemistry A 121 (2017): 7917. 28945363 10.1021/acs.jpca.7b08399

[39] R. Beck , A. Petrone , J. M. Kasper , M. J. Crane , P. J. Pauzauskie , and X. Li , “Effect of Surface Passivation on Nanodiamond Crystallinity,” Journal of Physical Chemistry C 122 (2018): 8573, 10.1021/acs.jpcc.8b00354.

[40] F. Coppola , P. Cimino , U. Raucci , M. G. Chiariello , A. Petrone , and N. Rega , “Exploring the Franck–Condon Region of a Photoexcited Charge Transfer Complex in Solution to Interpret Femtosecond Stimulated Raman Spectroscopy: Excited State Electronic Structure Methods to Unveil Non‐radiative Pathways,” Chemical Science 12 (2021): 8058. 34194695 10.1039/d1sc01238j PMC8208128

[41] F. Coppola , P. Cimino , F. Perrella , L. Crisci , A. Petrone , and N. Rega , “Electronic and Vibrational Manifold of Tetracyanoethylene–Chloronaphthalene Charge Transfer Complex in Solution: Insights From Td‐Dft and Ab Initio Molecular Dynamics,” Journal of Physical Chemistry. A 126 (2022): 7179. 36174118 10.1021/acs.jpca.2c05001 PMC9574931

[42] A. Petrone , F. Perrella , F. Coppola , et al., “Ultrafast Photo‐Induced Processes in Complex Environments: The Role of Accuracy in Excited‐State Energy Potentials and Initial Conditions,” Chemical Physics Reviews 3 (2022): 021307.

[43] X. Yin , X. Li , X. Li , et al., “Isomerization‐Induced Fluorescence Enhancement of Two New Viologen Derivatives: Mechanism Insight and Dft Calculations,” Chemical Science 14 (2023): 7016. 37389262 10.1039/d3sc02051g PMC10306075

[44] F. Coppola , P. Cimino , A. Petrone , and N. Rega , “Evidence of Excited‐State Vibrational Mode Governing the Photorelaxation of a Charge‐Transfer Complex,” Journal of Physical Chemistry A 128 (2024a): 1620. 38381887 10.1021/acs.jpca.3c08366

[45] J. Hafner , C. Wolverton , and G. Ceder , “Toward Computational Materials Design: The Impact of Density Functional Theory on Materials Research,” MRS Bulletin 31 (2006): 659, 10.1557/mrs2006.174.

[46] R. Beaulac , Y. Feng , J. W. May , E. Badaeva , D. R. Gamelin , and X. Li , “Orbital Pathways for Mn^2+^‐Carrier sp–d Exchange in Diluted Magnetic Semiconductor Quantum Dots,” Physical Review B 84 (2011): 195324.

[47] P. J. Lestrange , P. D. Nguyen , and X. Li , “Calibration of Energy‐Specific Tddft for Modeling k‐Edge Xas Spectra of Light Elements,” Journal of Chemical Theory and Computation 11 (2015): 2994, 10.1021/acs.jctc.5b00169. 26575736

[48] C. Daniel , “Photochemistry and Photophysics of Transition Metal Complexes: Quantum Chemistry,” Coordination Chemistry Reviews 282 (2015): 19.

[49] J. Aarons , M. Sarwar , D. Thompsett , and C.‐K. Skylaris , “Perspective: Methods for Large‐Scale Density Functional Calculations on Metallic Systems,” Journal of Chemical Physics 145 (2016): 220901, 10.1063/1.4972007. 27984887

[50] A. Petrone , J. J. Goings , and X. Li , “Quantum Confinement Effects on Optical Transitions in Nanodiamonds Containing Nitrogen Vacancies,” Physical Review B 94 (2016a): 165402, 10.1103/PhysRevB.94.165402.

[51] G. Donati , D. B. Lingerfelt , A. Petrone , N. Rega , and X. Li , “Watching Polaron Pair Formation From First‐Principles Electron‐Nuclear Dynamics,” Journal of Physical Chemistry. A 120 (2016): 7255, 10.1021/acs.jpca.6b06419. 27571540

[52] N. Li , Z. Zhu , C.‐C. Chueh , et al., “Mixed Cation FaP_𝓍_ea_1‐𝓍_Pbi_3_ With Enhanced Phase and Ambient Stability Toward High‐Performance Perovskite Solar Cells,” Advanced Energy Materials 7 (2016b): 1601307, 10.1002/aenm.201601307.

[53] D. C. Gary , S. E. Flowers , W. Kaminsky , A. Petrone , X. Li , and B. M. Cossairt , “Single‐Crystal and Electronic Structure of a 1.3 Nm Indium Phosphide Nanocluster,” Journal of the American Chemical Society 138 (2016): 1510, 10.1021/jacs.5b13214. 26784649

[54] D. C. Gary , A. Petrone , X. Li , and B. M. Cossairt , “Investigating the Role of Amine in Inp Nanocrystal Syntheses: Destabilizing Cluster Intermediates by z‐Type Ligand Displacement,” Chemical Communications 53 (2017): 161, 10.1039/C6CC07952K. 27904891

[55] J. L. Stein , M. I. Steimle , M. W. Terban , et al., “Cation Exchange Induced Transformation of Inp Magic‐Sized Clusters,” Chemistry of Materials 29 (2017): 7984, 10.1021/acs.chemmater.7b03075.

[56] G. Donati , D. B. Lingerfelt , C. M. Aikens , and X. Li , “Molecular Vibration Induced Plasmon Decay,” Journal of Physical Chemistry C 121 (2017): 15368, 10.1021/acs.jpcc.7b04451.

[57] F. Coppola , M. Hussain , J. Zhao , A. M. El‐Zohry , and M. Pastore , “Key Role of Electronic and Structural Properties in Regulating Intersystem Crossing: An In‐Depth Investigation on Naphthalene‐Diimide Triads for Thermally Activated Delayed Fluorescence Applications,” Journal of Physical Chemistry C 128 (2024b): 11998.

[58] S. Xu , J. E. Smith , S. Gozem , A. I. Krylov , and J. M. Weber , “Electronic Spectra of Tris (2, 2′‐Bipyridine)‐m (Ii) Complex Ions In Vacuo (m= Fe and Os),” Inorganic Chemistry 56 (2017): 7029. 28586198 10.1021/acs.inorgchem.7b00620

[59] U. Raucci , M. G. Chiariello , F. Coppola , et al., “An Electron Density Based Analysis to Establish the Electronic Adiabaticity of Proton Coupled Electron Transfer Reactions,” Journal of Computational Chemistry 41 (2020a): 1835. 32500950 10.1002/jcc.26224

[60] A. Petrone , D. B. Lingerfelt , N. Rega , and X. Li , “From Charge‐Transfer to a Charge‐Separated State: A Perspective From the Real‐Time Tddft Excitonic Dynamics,” Physical Chemistry Chemical Physics 16 (2014): 24457, 10.1039/C4CP04000G. 25306872

[61] G. Lever , D. J. Cole , R. Lonsdale , et al., “Large‐Scale Density Functional Theory Transition State Searching in Enzymes,” Journal of Physical Chemistry Letters 5 (2014): 3614, 10.1021/jz5018703. 26278727

[62] A. Petrone , D. B. Lingerfelt , D. B. Williams‐Young , and X. Li , “Ab Initio Transient Vibrational Spectral Analysis,” Journal of Physical Chemistry Letters 7 (2016b): 4501, 10.1021/acs.jpclett.6b02292. 27788583

[63] E. Battista , P. L. Scognamiglio , N. Di Luise , et al., “Turn‐On Fluorescence Detection of Protein by Molecularly Imprinted Hydrogels Based on Supramolecular Assembly of Peptide Multi‐Functional Blocks,” Journal of Materials Chemistry B 6 (2018): 1207. 32254181 10.1039/c7tb03107f

[64] A. Wildman , G. Donati , F. Lipparini , B. Mennucci , and X. Li , “Nonequilibrium Environment Dynamics in a Frequency‐Dependent Polarizable Embedding Model,” Journal of Chemical Theory and Computation 15 (2018): 43. 30512961 10.1021/acs.jctc.8b00836

[65] F. Perrella , U. Raucci , M. G. Chiariello , et al., “Unveiling the Structure of a Novel Artificial Heme‐Enzyme With Peroxidase‐Like Activity: A Theoretical Investigation,” Biopolymers 109 (2018): e23225. 30091460 10.1002/bip.23225

[66] G. Donati , A. Petrone , P. Caruso , and N. Rega , “The Mechanism of a Green Fluorescent Protein Proton Shuttle Unveiled in the Time‐Resolved Frequency Domain by Excited State Ab Initio Dynamics,” Chemical Science 9 (2018): 1126. 29675157 10.1039/c7sc02803b PMC5890789

[67] M. Marazzi , H. Gattuso , M. Fumanal , C. Daniel , and A. Monari , “Charge‐Transfer Versus Charge‐Separated Triplet Excited States of [Rei (Dmp)(co) 3 (his124)(trp122)]+ in Water and in Modified *pseudomonas aeruginosa* Azurin Protein,” Chemistry ‐ A European Journal 25 (2019): 2519. 30379366 10.1002/chem.201803685

[68] U. Raucci , F. Perrella , G. Donati , M. Zoppi , A. Petrone , and N. Rega , “Ab‐Initio Molecular Dynamics and Hybrid Explicit‐Implicit Solvation Model for Aqueous and Nonaqueous Solvents: Gfp Chromophore in Water and Methanol Solution as Case Study,” Journal of Computational Chemistry 41 (2020b): 2228. 32770577 10.1002/jcc.26384

[69] F. Coppola , F. Perrella , A. Petrone , G. Donati , and N. Rega , “A Not Obvious Correlation Between the Structure of Green Fluorescent Protein Chromophore Pocket and Hydrogen Bond Dynamics: A Choreography From Ab Initio Molecular Dynamics,” Frontiers in Molecular Biosciences 7 (2020): 569990. 33195416 10.3389/fmolb.2020.569990 PMC7653547

[70] M. E. Casida and M. Huix‐Rotllant , “Progress in Time‐Dependent Density‐Functional Theory,” Annual Review of Physical Chemistry 63 (2012): 287. 10.1146/annurev-physchem-032511-143803 22242728

[71] P.‐F. Loos , M. Boggio‐Pasqua , A. Scemama , M. Caffarel , and D. Jacquemin , “Reference Energies for Double Excitations,” Journal of Chemical Theory and Computation 15 (2019): 1939–1956. 30689951 10.1021/acs.jctc.8b01205

[72] Y. Horbatenko , S. Lee , M. Filatov , and C. H. Choi , “How Beneficial Is the Explicit Account of Doubly‐Excited Configurations in Linear Response Theory?,” Journal of Chemical Theory and Computation 17 (2021): 975. 33395286 10.1021/acs.jctc.0c01214

[73] R. Cammi , B. Mennucci , and J. Tomasi , “Fast Evaluation of Geometries and Properties of Excited Molecules in Solution: A Tamm‐Dancoff Model With Application to 4‐Dimethylaminobenzonitrile,” Journal of Physical Chemistry. A 104 (2000): 5631.

[74] M. Cossi and V. Barone , “Time‐Dependent Density Functional Theory for Molecules in Liquid Solutions,” Journal of Chemical Physics 115 (2001): 4708.

[75] S. Corni , R. Cammi , B. Mennucci , and J. Tomasi , “Electronic Excitation Energies of Molecules in Solution Within Continuum Solvation Models: Investigating the Discrepancy Between State‐Specific and Linear‐Response Methods,” Journal of Chemical Physics 123 (2005): 134512. 16223319 10.1063/1.2039077

[76] M. Caricato , B. Mennucci , J. Tomasi , et al., “Formation and Relaxation of Excited States in Solution: A New Time Dependent Polarizable Continuum Model Based on Time Dependent Density Functional Theory,” Journal of Chemical Physics 124 (2006): 124520. 16599710 10.1063/1.2183309

[77] R. Improta , V. Barone , G. Scalmani , and M. J. Frisch , “A State‐Specific Polarizable Continuum Model Time Dependent Density Functional Theory Method for Excited State Calculations in Solution,” Journal of Chemical Physics 125 (2006): 054103. 16942199 10.1063/1.2222364

[78] R. Improta , G. Scalmani , M. J. Frisch , and V. Barone , “Toward Effective and Reliable Fluorescence Energies in Solution by a New State Specific Polarizable Continuum Model Time Dependent Density Functional Theory Approach,” Journal of Chemical Physics 127 (2007): 074504. 17718617 10.1063/1.2757168

[79] J. D. Gaynor , J. Sandwisch , and M. Khalil , “Vibronic Coherence Evolution in Multidimensional Ultrafast Photochemical Processes,” Nature Communications 10 (2019): 5621. 10.1038/s41467-019-13503-9 PMC6901526 31819052

[80] J. D. Gaynor , R. B. Weakly , and M. Khalil , “Multimode Two‐Dimensional Vibronic Spectroscopy. i. Orientational Response and Polarization‐Selectivity,” Journal of Chemical Physics 154 (2021): 184201. 34241026 10.1063/5.0047724

[81] V. Barone and M. Cossi , “Quantum Calculation of Molecular Energies and Energy Gradients in Solution by a Conductor Solvent Model,” Journal of Physical Chemistry. A 102 (1998): 1995–2001.

[82] M. Cossi , G. Scalmani , N. Rega , and V. Barone , “New Developments in the Polarizable Continuum Model for Quantum Mechanical and Classical Calculations on Molecules in Solution,” Journal of Chemical Physics 117 (2002): 43.

[83] M. Cossi , N. Rega , G. Scalmani , and V. Barone , “Energies, Structures, and Electronic Properties of Molecules in Solution With the c‐Pcm Solvation Model,” Journal of Computational Chemistry 24 (2003): 669. 12666158 10.1002/jcc.10189

[84] T. Yanai , D. P. Tew , and N. C. Handy , “A New Hybrid Exchange–Correlation Functional Using the Coulomb‐Attenuating Method (Cam‐b3lyp),” Chemical Physics Letters 393 (2004): 51.

[85] M. J. Peach , P. Benfield , T. Helgaker , and D. J. Tozer , “Excitation Energies in Density Functional Theory: An Evaluation and a Diagnostic Test,” Journal of Chemical Physics 128 (2008): 044118. 18247941 10.1063/1.2831900

[86] A. D. Dwyer and D. J. Tozer , “Effect of Chemical Change on Tddft Accuracy: Orbital Overlap Perspective of the Hydrogenation of Retinal,” Physical Chemistry Chemical Physics 12 (2010): 2816. 20449370 10.1039/c002428g

[87] T. Clark , J. Chandrasekhar , G. W. Spitznagel , and P. V. R. Schleyer , “Efficient Diffuse Function‐Augmented Basis Sets for Anion Calculations. Iii. The 3‐21+ g Basis Set for First‐Row Elements, li–f,” Journal of Computational Chemistry 4 (1983): 294.

[88] M. J. Frisch , J. A. Pople , and J. S. Binkley , “Self‐Consistent Molecular Orbital Methods 25. Supplementary Functions for Gaussian Basis Sets,” Journal of Chemical Physics 80 (1984): 3265.

[89] S. Grimme , “Semiempirical Gga‐Type Density Functional Constructed With a Long‐Range Dispersion Correction,” Journal of Computational Chemistry 27 (2006): 1787. 16955487 10.1002/jcc.20495

[90] S. Grimme , J. Antony , S. Ehrlich , and H. Krieg , “A Consistent and Accurate Ab Initio Parametrization of Density Functional Dispersion Correction (Dft‐d) for the 94 Elements h‐Pu,” Journal of Chemical Physics 132 (2010): 154104. 20423165 10.1063/1.3382344

[91] S. Grimme , S. Ehrlich , and L. Goerigk , “Effect of the Damping Function in Dispersion Corrected Density Functional Theory,” Journal of Computational Chemistry 32 (2011): 1456. 21370243 10.1002/jcc.21759

[92] M. J. Frisch , G. W. Trucks , H. B. Schlegel , et al., Gaussian1̃6 Revision C.01 (Wallingford CT: Gaussian Inc, 2016).

[93] L. Serrano‐Andrés and M. Merchán , “Quantum Chemistry of the Excited State: 2005 Overview,” Journal of Molecular Structure: THEOCHEM 729 (2005): 99.

[94] B. O. Roos , “Multiconfigurational Quantum Chemistry,” in Theory and Applications of Computational Chemistry (Amsterdam, Netherlands: Elsevier, 2005), 725–764.

[95] L. Gagliardi and B. O. Roos , “Multiconfigurational Quantum Chemical Methods for Molecular Systems Containing Actinides,” Chemical Society Reviews 36 (2007): 893. 17534476 10.1039/b601115m

[96] C. Angeli , R. Cimiraglia , and M. Pastore , “A Comparison of Various Approaches in Internally Contracted Multireference Configuration Interaction: The Carbon Dimer as a Test Case,” Molecular Physics 110 (2012): 2963.

[97] M. Pastore , F. De Angelis , and C. Angeli , “Optical Absorption Spectrum of the n3 Solar Cell Sensitizer by Second‐Order Multireference Perturbation Theory,” Theoretical Chemistry Accounts 135 (2016): 1.

[98] J. Olsen , “The Casscf Method: A Perspective and Commentary,” International Journal of Quantum Chemistry 111 (2011): 3267.

[99] S. Larsson , “Applications of Casscf,” International Journal of Quantum Chemistry 111 (2011): 3424.

[100] K. Andersson , P.‐Å. Malmqvist , and B. O. Roos , “Second‐Order Perturbation Theory With a Complete Active Space Self‐Consistent Field Reference Function,” Journal of Chemical Physics 96 (1992): 1218.

[101] E. Bignon , M. Marazzi , V. Besancenot , et al., “Ibuprofen and Ketoprofen Potentiate Uva‐Induced Cell Death by a Photosensitization Process,” Scientific Reports 7 (2017): 8885. 28827702 10.1038/s41598-017-09406-8 PMC5566383

[102] M. Nucci , A. Núñez , L. M. Frutos , and M. Marazzi , “Design of Improved Molecular Solar‐Thermal Systems by Mechanochemistry: The Case of Azobenzene,” Advanced Sustainable Systems 6 (2022): 2200097.

[103] F. Coppola , M. Nucci , M. Marazzi , D. Rocca , and M. Pastore , “Norbornadiene/Quadricyclane System in the Spotlight: The Role of Rydberg States and Dynamic Electronic Correlation in a Solar‐Thermal Building Block,” ChemPhotoChem 7 (2023): e202200214.

[104] S. Battaglia , I. F. Galván , and R. Lindh , Theoretical and Computational Photochemistry (Amsterdam, Netherlands: Elsevier, 2023), 135–162.

[105] G. Li Manni , I. Fdez , A. Galván , et al., “The Openmolcas Web: A Community‐Driven Approach to Advancing Computational Chemistry,” Journal of Chemical Theory and Computation 19 (2023): 6933. 37216210 10.1021/acs.jctc.3c00182 PMC10601490

[106] R. Kobayashi and R. D. Amos , “The Application of Cam‐b3lyp to the Charge‐Transfer Band Problem of the Zincbacteriochlorin–Bacteriochlorin Complex,” Chemical Physics Letters 420 (2006): 106.

[107] Z.‐L. Cai , M. J. Crossley , J. R. Reimers , R. Kobayashi , and R. D. Amos , “Density Functional Theory for Charge Transfer: The Nature of the n‐Bands of Porphyrins and Chlorophylls Revealed Through Cam‐b3lyp, caspt2, and Sac‐Ci Calculations,” Journal of Physical Chemistry. B 110 (2006): 15624. 16884287 10.1021/jp063376t

[108] I. V. Rostov , R. D. Amos , R. Kobayashi , G. Scalmani , and M. J. Frisch , “Studies of the Ground and Excited‐State Surfaces of the Retinal Chromophore Using Cam‐b3lyp,” Journal of Physical Chemistry. B 114 (2010): 5547. 20369810 10.1021/jp911329g

[109] A. Petrone , J. Cerezo , F. J. A. Ferrer , et al., “Absorption and Emission Spectral Shapes of a Prototype Dye in Water by Combining Classical/Dynamical and Quantum/Static Approaches,” Journal of Physical Chemistry. A 119 (2015): 5426. 25699575 10.1021/jp510838m

[110] J. Cerezo , A. Petrone , F. J. A. Ferrer , et al., “Electronic Spectroscopy of a Solvatochromic Dye in Water: Comparison of Static Cluster/Implicit and Dynamical/Explicit Solvent Models on Structures and Energies,” Theoretical Chemistry Accounts 135 (2016): 1.

[111] M. Savarese , A. Aliberti , I. De Santo , et al., “Fluorescence Lifetimes and Quantum Yields of Rhodamine Derivatives: New Insights From Theory and Experiment,” Journal of Physical Chemistry. A 116 (2012): 7491. 22667332 10.1021/jp3021485

