# Supplementary material for: A Cost‐Effective Computational Strategy for the Electronic Layout Characterization of a Second Generation Light‐Driven Molecular Rotary Motor in Solution
Source: J Comput Chem. 2025 Jan 11;46(2):e70023. doi: 10.1002/jcc.70023 (PMC11724392; doi:10.1002/jcc.70023)
Supplement: Supplementary file 1 — Supplementary Data S1. [file JCC-46-0-s001.pdf]

Supporting Information: A cost-effective computational strategy for the electronic layout characterization of a second generation light-driven molecular rotary motor in solution†

Raoul Carfora+,†,‡ Federico Coppola+,† Paola Cimino,‡ Alessio Petrone,*,‡,†,¶ and Nadia Rega*,‡,†,¶

†*Scuola Superiore Meridionale, Largo San Marcellino 10, I-80138, Napoli, Italy.*

‡*Department of Chemical Sciences, University of Napoli Federico II, Complesso Universitario di M.S. Angelo, via Cintia 21, I-80126, Napoli, Italy.*

¶*Istituto Nazionale Di Fisica Nucleare, sezione di Napoli, Complesso Universitario di M.S. Angelo ed. 6, via Cintia 21, I-80126, Napoli, Italy.*

E-mail: alessio.petrone@unina.it; nadia.rega@unina.it

+*Both authors contributed equally to this work.*

Contents

Fig. S1: Active Space employed for CASPT2//SA(8)CASSCF/6-31G(d,p) calculations

Tab. S1: Description of excited states at CASSCF, SS-CASPT2 and MS-CASPT2 levels of theory

Fig. S2: Distorted geometry structure, with the corresponding torsion and pyramidalization angle values

Tab. S2: TD-DFT calculations for the distorted geometry and for the Min2

Tab. S3: MS-CASPT2 and CASSCF excited state calculations for the distorted geometry

Fig. S3: Composition of Normal Modes of vibration

Tabs. S4 and S5: Cartesian coordinates of the Min1 and Min2

S1 CASSCF and MS-CASPT2 additional computational details

The default IPEA shift (0.25 E_h) and an imaginary shift of 0.25 a.u. has been applied to the zeroth-order Hamiltonian to remove eventual intruder states. The RAS State Interaction method has been invoked for the calculation of excited state properties such as oscillator strength (f) and transition dipole moment (TDM). Oscillator strength at MS-CASPT2 level have been estimated as: f=2/3$(TDM_{CAS})^2 \Delta E_{PT2}$, considering the TDMs computed at CASSCF level and perturbed ΔE in atomic units.

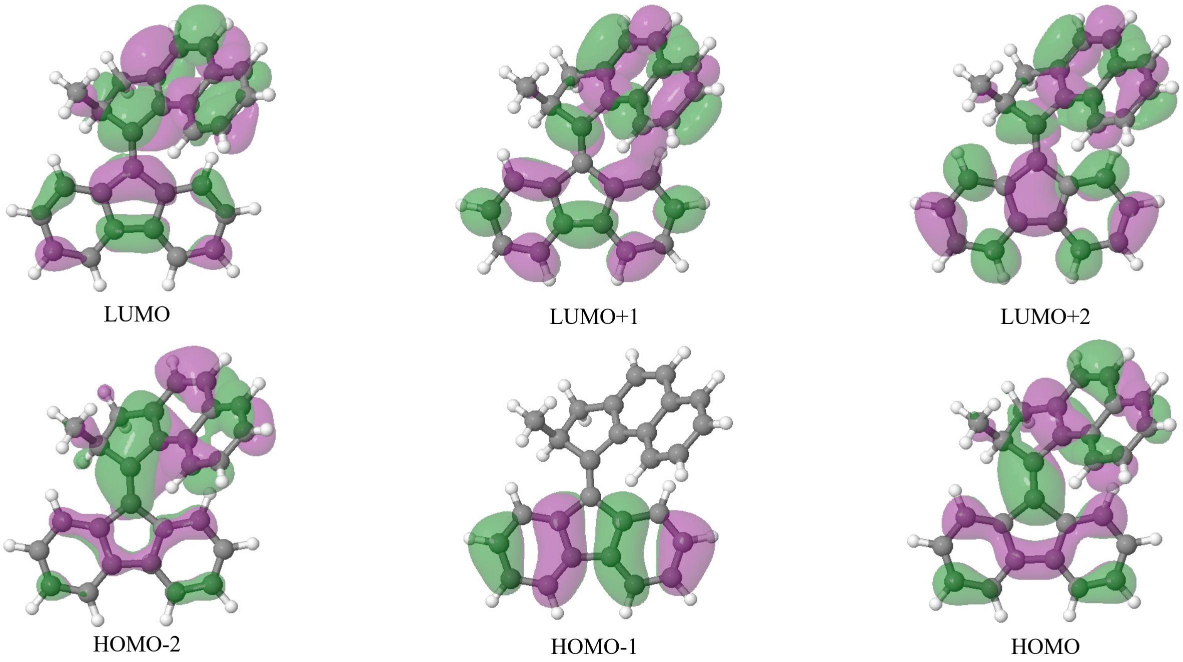

Figure S1. Active Space composed by 6 electrons in 6 molecular orbitals (iso: 0.02) employed for the calculation of CASPT2//SA(8)CASSCF/6-31G(d,p) vertical excitation energies and related properties.

Table S1. Vertical excitation energies (VEE, eV) at CASSCF(6,6), single state (SS) and multistate (MS) CASPT2 levels and principal configuration (χ) for the first seven singlet states. Oscillator strengths, f dimensionless, are reported for CASSCF and MS-CASPT2 level. Molecular orbital representations are reported in Fig. S1. All values were obtained using the Min1 optimized geometry at B3LYP/6-31G(d,p)/C-PCM in cyclohexane.

	SA(8)-CASSCF(6,6)	χ	f	SS-CASPT2	χ	MS-CASPT2	χ	f
$S_1 \leftarrow S_0$	4.49	HOMO-1-LUMO	0.02	3.70	HOMO-LUMO	3.49	HOMO-LUMO	0.77
$S_2 \leftarrow S_0$	4.99	HOMO-LUMO	1.04	3.71	HOMO-1-LUMO	3.72	HOMO-1-LUMO	0.01
$S_3 \leftarrow S_0$	5.41	$(\text{HOMO-LUMO})_2$	0.28	4.43	$(\text{HOMO-LUMO})_2$	4.57	$(\text{HOMO-LUMO})_2$	0.24
$S_4 \leftarrow S_0$	6.30	HOMO-LUMO+2	0.08	4.91	HOMO-2-LUMO	4.92	HOMO-2-LUMO	0.06
$S_5 \leftarrow S_0$	6.30	HOMO-LUMO+1	1.07	4.97	HOMO-LUMO+2	5.04	HOMO-LUMO+1	0.85
$S_6 \leftarrow S_0$	6.81	HOMO-1-LUMO+2	0.25	5.09	HOMO-LUMO+1	5.08	HOMO-LUMO+2	0.06
$S_7 \leftarrow S_0$	7.35	HOMO-2-LUMO	0.08	5.63	HOMO-1-LUMO+2	5.76	HOMO-1-LUMO+2	0.18

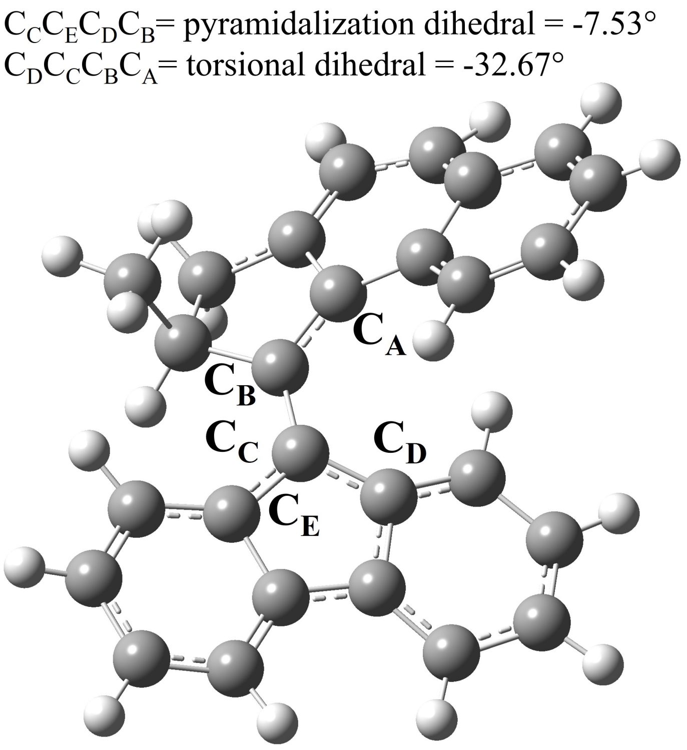

Figure S2. Chosen structure, namely γ, that is distorted along the two main coordinates involved in the photoisomerizaion, the dihedral angle $C_DC_CC_BC_A$ related to the torsion of the rotor respect to the stator and the dihedral angle $C_CC_EC_DC_B$ related to the pyramidalization of the carbon C_C. Such distortions are obtained starting from the Min1 and following the geometrical distortion observed in Ref. S1 and S2. Values of the two dihedral angles are reported. This structure corresponds to the Franck-Condon region near the Min1, according the accurate energy analysis presented in Figure 4 of Ref. S1.

Table S2. TD-CAM-B3LYP/6-31G(d,p) vertical excitation energies (in eV) and oscillator strengths (dimensionless and in parenthesis) of the $S_1 \leftarrow S_0$ and $S_2 \leftarrow S_0$ electronic transitions, for the γ (see Fig. S2) and Min2 (see main article) structures. The corresponding energy separation (Δ S_2-S_1, in eV) between S_2 and S_1 is also reported in the last column.

	$S_1 \leftarrow S_0$	$S_2 \leftarrow S_0$	ΔS_2-S_1
Geo γ	2.48 (0.52)	3.04 (0.00)	0.56
Min2	3.10 (0.50)	3.55 (0.00)	0.45

Table S3. MS-CASPT2//SA(8)CASSCF(6,6)/6-31G(d,p) gas-phase low-lying excited characterization for the γ structure (see Fig. S2). VEEs in eV, oscillator strengths (f, dimensionless), and χ (the main MO contribution to the transitions).

Geo γ	VEE	f	χ
$S_1 \leftarrow S_0$	2.34	0.17	HOMO-LUMO
$S_2 \leftarrow S_0$	3.02	0.01	HOMO-1-LUMO

S2 Normal modes of vibration

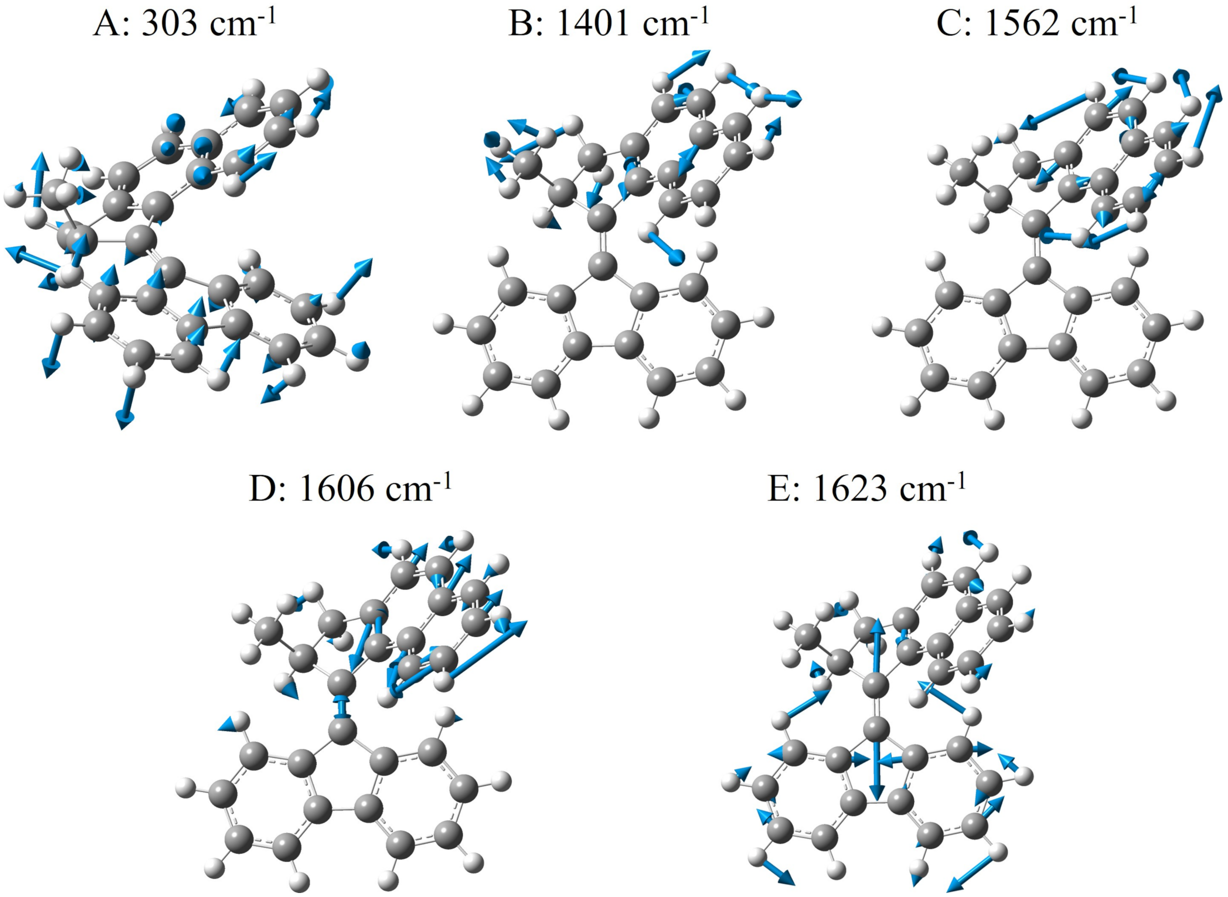

Figure S3. Normal mode displacement vectors computed in ground state at B3LYP/6-31G(d,p)/C-PCM cyclohexane level of theory. See also Tab. 5 in the main text for details.

S3 Equilibrium geometries in cartesian coordinates (Å) at B3LYP/6-31G(d,p)/C-PCM(C_6H_{12})

Table S4. Minimum energy structure: Min1

C	5.15442400	-0.83600600	0.73313100
C	4.59031400	0.38531200	0.36045500
C	3.22577200	0.44526000	0.08181900
C	2.40051000	-0.70672000	0.16715600
C	2.98052700	-1.91798900	0.56481200
C	4.35094900	-1.97591500	0.83531500
H	6.21639200	-0.89951100	0.95117000
H	5.20748500	1.27678000	0.29218600
H	2.39316100	-2.81901300	0.67808900
H	4.79300600	-2.92180800	1.13443100
C	2.39873300	1.57884400	-0.32686200
C	2.76184600	2.88954600	-0.63466000
C	1.78528200	3.77106000	-1.10119200
C	0.46977500	3.32957000	-1.27993500
C	0.10134300	2.01823200	-0.96750300
C	1.05749700	1.13503300	-0.45296100
H	3.79276600	3.21652300	-0.53154300
H	2.05128300	4.79533400	-1.34538800
H	-0.27889200	4.01211900	-1.67138700
H	-0.91851500	1.70124900	-1.13829300
C	0.99487000	-0.31514500	-0.12735500
C	-1.53037700	-0.82921100	-0.25612500
C	-2.31360800	0.18598200	0.38585300
C	-3.72208600	0.22312000	0.10030600
C	-4.29863800	-0.75605700	-0.75534500
C	-3.54201400	-1.78615800	-1.27120900
C	-2.16314000	-1.83186400	-0.99160800
H	-4.00309200	-2.56091800	-1.87725600
C	-1.17840400	-2.91161600	-1.36429600
C	-0.00919800	-2.66632200	-0.36462800
C	-0.09145800	-1.14898300	-0.16610300
H	-1.60421100	-3.91743700	-1.28231100
H	-0.82595800	-2.78920300	-2.39715300
H	0.94494900	-2.94949400	-0.81371400
C	-0.23047300	-3.46503800	0.93473200
H	0.51276200	-3.21727400	1.69755300
H	-1.21863400	-3.25194600	1.35451800
H	-0.17040100	-4.54044000	0.73626000
C	-1.79657700	1.10224900	1.33972200
C	-2.61173700	2.03728500	1.94072900
C	-3.98681100	2.11001600	1.61661200
C	-4.52769300	1.21684500	0.71990500
H	-0.74667400	1.05187300	1.60092200
H	-2.19537900	2.72260000	2.67304400
H	-4.61474100	2.85929300	2.08918400
H	-5.58856100	1.24547800	0.48548100
H	-5.36450800	-0.70218000	-0.96037900

nuclear repulsion energy 2265.790630 Hartrees

Table S5. Minimum energy structure: Min2

C	-1.73724000	3.53583400	-1.63812700
C	-2.68420200	2.76388100	-0.96296100
C	-2.31146600	1.51377400	-0.46884000
C	-0.98710800	1.02661100	-0.62731600
C	-0.06156000	1.79507900	-1.34368600
C	-0.43987800	3.04646800	-1.83457600
H	-2.01189100	4.51184900	-2.02731500
H	-3.70103600	3.12633200	-0.83958800
H	0.94255100	1.43113800	-1.52475600
H	0.28393500	3.64525700	-2.37977600
C	-3.10179400	0.48660600	0.20702400
C	-4.41478300	0.51412600	0.67691500
C	-4.91592100	-0.59720400	1.35730700
C	-4.09898000	-1.71147200	1.58573900
C	-2.78262500	-1.74228800	1.12005600
C	-2.27961600	-0.65598600	0.39260500
H	-5.03602100	1.39342900	0.53070800
H	-5.93684000	-0.59053900	1.72751500
H	-4.48933200	-2.56084600	2.13871700
H	-2.16300100	-2.60509700	1.33841200
C	-0.91804500	-0.36080500	-0.10854500
C	1.58824600	-0.90662600	-0.10764700
C	2.28657800	0.21481200	0.47098900
C	3.72058400	0.23748700	0.36429700
C	4.40899300	-0.85474600	-0.23293900
C	3.72896300	-1.97301000	-0.65967000
C	2.32464200	-1.99821400	-0.57250700
H	4.26540500	-2.82914700	-1.05879800
C	1.42589600	-3.14626800	-0.92346600
C	0.03678600	-2.75091600	-0.33975400
C	0.15433300	-1.22578600	-0.14243600
H	1.34835000	-3.26430300	-2.01300600
H	1.79671900	-4.09966700	-0.53166000
H	-0.06786500	-3.24052300	0.63984300
C	-1.08394400	-3.23013600	-1.27325700
H	-2.08362800	-3.03947100	-0.88558600
H	-1.00161200	-2.73509800	-2.24643900
H	-0.98719200	-4.30911400	-1.43645500
C	1.66278200	1.25327800	1.21012700
C	2.39802700	2.28639700	1.75387700
C	3.79930500	2.34364500	1.58381500
C	4.44426100	1.33175000	0.90951500
H	0.59274800	1.22333900	1.36283500
H	1.89312200	3.06091200	2.32354800
H	4.36400400	3.17057300	2.00365400
H	5.52575200	1.34205000	0.80269000
H	5.49242500	-0.80883200	-0.30377100

nuclear repulsion energy 2269.586060 Hartrees

References

[S1] Pang, X.; Cui, X.; Hu, D.; Jiang, C.; Zhao, D.; Lan, Z.; Li, F. "Watching" the Dark State in Ultrafast Nonadiabatic Photoisomerization Process of a Light-Driven Molecular Rotary Motor. *J. Phys. Chem. A* **2017**, *121*, 1240–1249.

[S2] Roy, P.; Sardjan, A. S.; Browne, W. R.; Feringa, B. L.; Meech, S. R. Excited state dynamics in unidirectional photochemical molecular motors. *J. Am. Chem. Soc.* **2024**, *146*, 12255–12270.
